# *Kirkegaardia* Blake, 2016 (Annelida: Cirratulidae) from Southeastern Brazil with description of nine new species

**DOI:** 10.1371/journal.pone.0265336

**Published:** 2022-05-10

**Authors:** Roberta Freitas, Rannyele Passos Ribeiro, Christine Ruta

**Affiliations:** 1 Departamento de Zoologia, Universidade Federal do Rio de Janeiro, Rio de Janeiro, Brazil; 2 Washington University in St. Louis, St. Louis, Missouri, United States of America; 3 Marine Biological Laboratory, Woods Hole, MA, United States of America; CIIMAR Interdisciplinary Centre of Marine and Environmental Research of the University of Porto, PORTUGAL

## Abstract

This is the first taxonomic study of cirratulid polychaetes of the genus *Kirkegaardia* Blake, 2016 from Brazil. Nine new species of the genus are described from the Southern Brazilian coast (50–3000 m deep). The genus *Kirkegaardia* is generally subdivided into three distinct groups of species (*Kirkegaardia dorsobranchialis*-*heterochaeta*, *Kirkegaardia baptisteae*-*tesselata* and *Kirkegaardia luticastella*) and several out-group species for which relationships remains to be defined. In this study, new species were included in the *Kirkegaardia dorsobranchialis*-*heterochaeta* and *Kirkegaardia baptisteae*-*tesselata* groups. *Kirkegaardia dorsobranchialis*-*heterochaeta* is characterized by thoracic parapodia elevated producing a channel between the notopodia, elongate pre-setigerous region that is either entirely smooth or modified with a dorsal ridge and/or rings, and noto- and neurosetae capillaries denticulated. As belonging to this group, *K*. *blakei* sp. nov., *K*. *brisae* sp. nov., *K*. *goytaca* sp. nov., *K*. *jongo* sp. nov. and *K*. *papaveroi* sp. nov. are described here. *Kirkegaardia baptisteae*-*tesselata* includes species that lack thoracic parapodia elevated and mid-dorsal thoracic groove, although a dorsal ridge is sometimes developed. In the pre-setigerous region dorsal ridges and rings are present or absent. Most species in this group have neurosetae denticulated, and notosetae capillaries of other types. This study adds *K*. *helenae* sp. nov., *K*. *medusa* sp. nov., *K*. *nupem* sp. nov. and *K*. *zafirae* sp. nov. to the latter species group. In addition, two new records are provided for *K*. *hampsoni*. A key to cirratulid polychaete species reported from Brazilian waters is provided.

## Introduction

Cirratulidae Ryckholt, 1851 [1] is a family of sedentarian annelids that are recognized by the numerous tentacles and paired filamentous branchiae located on the anterior segments [2]. The family Cirratulidae comprises 21 genera and 291 species described [3]. Cirratulids are deposit feeding organisms, mostly small (measuring about 2.5 cm long) and inhabit all oceans from the intertidal to the deep sea in consolidated and unconsolidated sediments [4, 5]. As deposit feeders, some cirratulids have been reported as bio-indicators of organic enrichment in studies of marine environmental assessment [6–8]. Cirratulids are also interesting organisms for studies on annelid reproduction because they show different modes of sexual and asexual reproduction, including parthenogenesis and viviparity [9–11]. Also, they exhibit a high capacity of regeneration [11–15].

The taxonomy of cirratulids is challenging because the specimens have few morphological characters that are often lost during collection and manipulation, which makes describing new species difficult [16–18]. Bitentaculate cirratulids are not well known in Brazil [19, 20], as only 28 species have been registered up to date, according to the most recent species compilation [21]. The genus *Kirkegaardia* Blake, 2016 [22] has not been recorded from Brazil. *Kirkegaardia* is a recently described genus that includes many synonymized species of other genera; for example, *Kirkegaardia dorsobranchialis* (Kirkegaard, 1959) [23], *Kirkegaardia heterochaeta* (Laubier, 1961) [24], and *Kirkegaardia secunda* (Banse & Hobson, 1968) [25], which were originally described in the genus *Tharyx* Webster & Benedict, 1887 [26].

*Kirkegaardia* is characterized by having a long and thin body with slightly expanded anterior and thoracic segments; a pair of dorsal tentacles inserted in the final portion of the peristomium or in the first thoracic setiger; usually with several pairs of branchiae, beginning from the peristomium; and denticulate or serrated capillaries. Species of *Kirkegaardia* (and earlier as *Monticellina*) were recorded in both the Northern [2, 27] and Southern Atlantic Ocean [28]. In South America, the genus has only been recorded for Argentina, from which *Kirkegaardia morae* (Elias, Rivero & Orensanz, 2017) [28] was described.

This study represents the first records of *Kirkegaardia* for Brazil, with the description of nine new species collected from the Campos Basin, Southeastern Brazil (13–3000 m depth), namely *K*. *blakei* sp. nov., *K*. *brisae* sp. nov., *K*. *helenae* sp. nov., *K*. *goytaca* sp. nov., *K*. *jongo* sp. nov., *K*. *medusa* sp. nov., *K*. *nupem* sp. nov., *K*. *papaveroi* sp. nov. and *K*. *zafirae* sp. nov. In addition, this study expands the distribution of *K*. *hampsoni* Blake, 2016 to the Southern Atlantic [22].

## Material and methods

**Fig 1:**
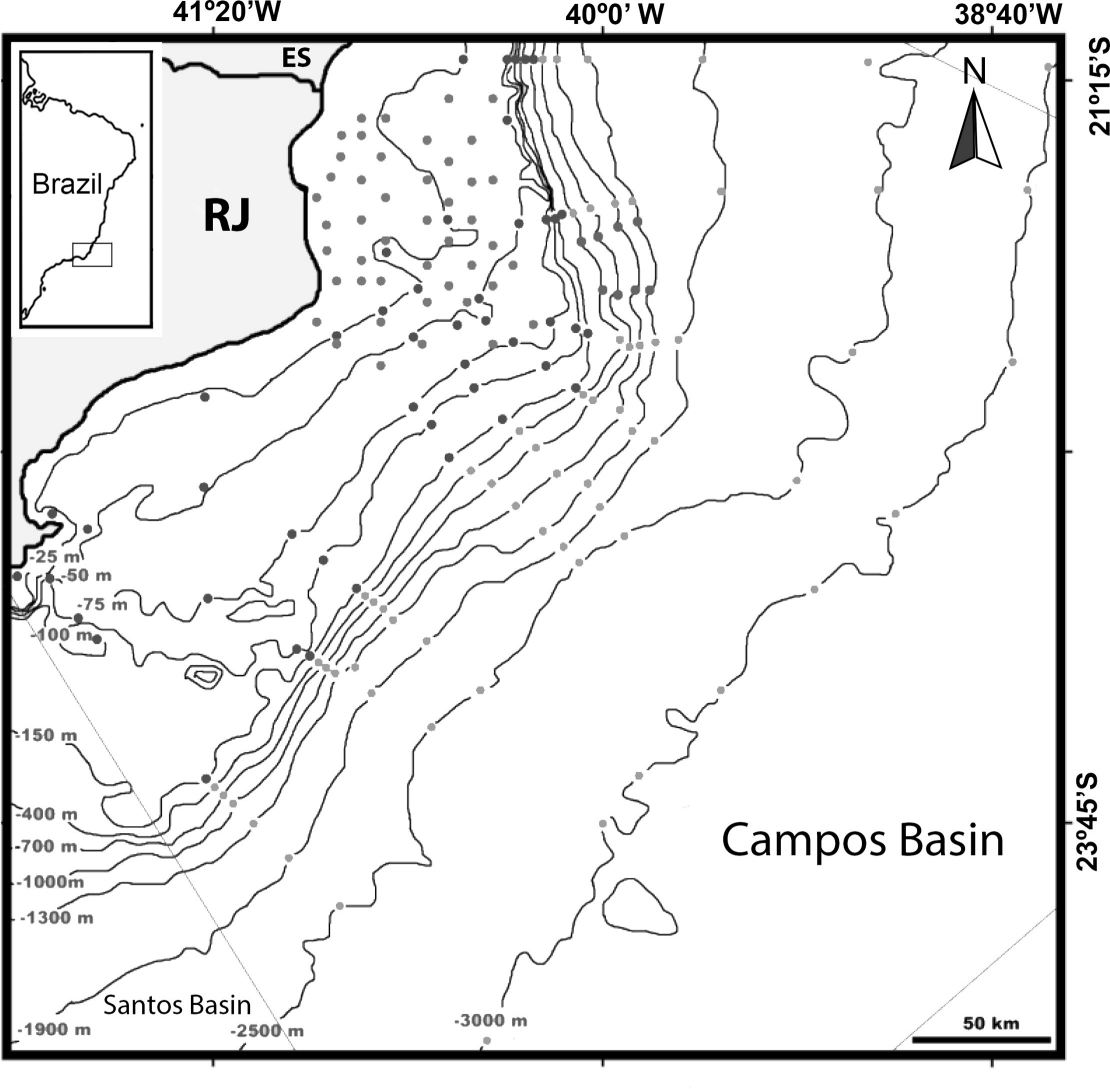
Study area, Campos Basin, Brazil. Sampling stations are represented by coloured dots.

The Campos Basin is bordered to the south by the top of Cabo Frio, which separates it from the Santos Basin; to the north by the top of Vitória, which separates it from the Espírito Santo Basin; and to the west by Precambrian rocks, which emerge near the city of Campos dos Goytacazes (RJ) (Fig 1) [29]. The region is characterized by the low river input and coastal and shelf upwelling of cold, nutrient-rich South Atlantic Central Water. These events are enhanced during austral spring and summer seasons under prevailing NE winds on the Cabo Frio coast (23S/42W), but also can occur intermittently all year round and reach hundreds of miles on the platform [30, 31].

The material analyzed for the present study came from two independent projects carried out in Southeastern Brazil, under the coordination of the research center CENPES/PETROBRAS (Petrobras Research Center): HABITATS–Environmental Heterogeneity in the Campos Basin (from 400 to 3000 m depth) and AMBES–Environmental Heterogeneity in the Espírito Santo Basin and northern region of the Campos Basin (from 50 to 3000 m depth). All permits required to conduct sampling are held by CENPES/PETROBRAS. The sediment was collected with a 0.25 m^2^ box-corer (Ocean Instruments, USNEL Spade Corer MK I). Samples were separated into three strata (0–2 cm, 2–5 cm, and 5–10 cm) and sieved with 0.3–0.5 mm meshes. The collected specimens were immediately preserved in 10% formalin, and later rinsed in fresh water and finally stored in 70% ethanol. Identifications were based on morphological characters. Specimens were examined under a stereomicroscope, compound light microscope, and scanning electron microscope (SEM). Specimens used for SEM were first dehydrated in a series of progressively increasing concentrations of ethanol (70–100%), critical point dried, covered with ~25 nm of gold, and then examined and photographed using the Electron Microscopy Laboratory (Protozoology, UFRJ). All specimens were measured using the ZEN 2012 blue version program from images taken with a camera attached to a Zeiss Primo Star optical microscope. Additionally, some specimens were stained with methyl green to investigate species-specific staining patterns. Type specimens and paratypes of the nine newly described species were deposited in the Polychaete Collection of the Rio de Janeiro National Museum-MNRJ / UFRJ, RJ, Brazil.

The following abbreviations are used here: br, branchia; dCr, dorsal crest; dGr, dorsal groove; neP, neuropodium; noP, notopodium; nuO, nuchal organ; per, peristomium; perC, peristomial crest; pr, prostomium; pyg, pygidium; tGr, thoracic groove; tn, tentacle; and vGr, ventral groove.

### Nomenclatural acts

The electronic edition of this article conforms to the requirements of the amended International Code of Zoological Nomenclature. Hence, the new names contained herein are available under that Code from the electronic edition of this article. This published work and the nomenclatural acts are registered in ZooBank. The ZooBank LSIDs (Life Science Identifiers) can be resolved and the associated information viewed through any standard web browser by appending the LSID to the prefix ″http://zoobank.org/″. The LSID for this publication is: urn:lsid:zoobank.org:pub: 7297C752-D78C-4A6D-B795-B870A099AFE5. The electronic edition of this work was published in a journal with an ISSN, and has been archived and is available from the following digital repositories: LOCKSS [author to insert any additional repositories].

## Results

### Systematics

#### Family Cirratulidae Ryckholt, 1851

Diagnosis. Body elongated, cylindrical, region anterior and/or posterior segments sometimes expanded. Thoracic region often expanded, abdominal region narrow or moniliform, sometimes expanded in the pre-pygidial region. Prostomium narrow and conical or wide, without appendages, with or without eyes. Peristomium achaetous, with or without rings. One or more pairs of tentacles located on the segmental groove that separates the peristomium from the first setiger or on the thoracic setigers. Dorsal tentacles grooved arise as single pairs or as multiple groups of filaments and cylindrical branchiae. Branchiae long, inserted dorsally at the base of the notopodium, usually present up to the abdominal region. Pharynx ventral, unarmed. Parapodia reduced, bi-ramous, with rudimentary lobes. Many epidermal simple chaetae, such as capillaries, simple hooks, bidentate or multidentate. Pygidium with a simple lobe, sometimes with sub-anal disk, or terminal cirrus [2, 32].

#### Genus *Kirkegaardia* Blake, 2016

Type species: *Monticellina heterochaeta* (Laubier 1961) [24]

Type locality. Banyuls-sur-Mer (France).

Diagnosis. Bitentaculate, with very distinct body regions. Prostomium short without annulations. Peristomium usually long and cylindrical, with annulations. Dorsal tentacles located in the final portion of the peristomium. Thoracic parapodia with inflated notopodia forming dorsal sulcus in the thoracic region or thoracic parapodia inflated, leaving the dorsal region as a crest. Parapodia of the non-inflated abdominal region laterally positioned. Posterior segments usually expanded or enlarged. Pre-pygidial abdominal segments wider than long and often expanded. Setae capillaries with distinct smooth (denticulate) edge, often basally expanded.

Remarks. The first species of *Kirkegaardia* was described as *Monticellina heterochaeta* Laubier, 1961 [24]. In 1966, Laubier assigned the genus *Monticellina* Laubier, 1961 [24] as a junior synonym of *Tharyx* Webster & Benedict, 1887 [26], a genus that was thought to have only capillary setae (Laubier, 1966) [33]. However, *Monticellina* was reestablished by Blake [27] for species with capillaries having a distinct serrated (denticulate) edge. Blake [27] also determined that *Tharyx acutus* Webster & Benedict, 1887 [26], the type-species, has sub-bidentate hooks, thus redefining the genus *Tharyx*, and removed species having all serrated capillaries to *Monticellina* and smooth capillaries to a new genus *Aphelochaeta*. Later, Blake [22] discovered that *Monticellina* was a junior homonym of the turbellarian genus *Monticellina* Westblad, 1953 [34], and renamed the polychaete genus *Kirkegaardia* to replace the homonym. Blake [22] also described 16 new species, increasing the number of *Kirkegaardia* species to 38.

### *Kirkegaardia hampsoni* Blake, 2016

Fig 2

*Tharyx dorsobranchialis*: Maciolek-Blake *et al*., 1985 [35]: 75, 142, Appendix B-3, Appendix D-8

*Monticellina dorsobranchialis*: Blake 1991 [27] (In part); Hilbig & Blake 2000 [36]: 162. Not Kirkegaard 1959.

*Kirkegaardia hampsoni*: Blake 2016 [22]: p. 28, figs. 12–13.

Material examined. BRAZIL: Campos Basin–-19.62828611°S -39.59208889°W, 35 m, 15/07/11, one ind., (MNRJP-002966); -19.58677500°S -39.64254444°W, 29 m, 12/12/10, two ind., (MNRJP-002967); -19.87490556°S -39.81891667°W, 42 m, 16/07/11, one ind., (MNRJP-002968).

**Fig 2:**
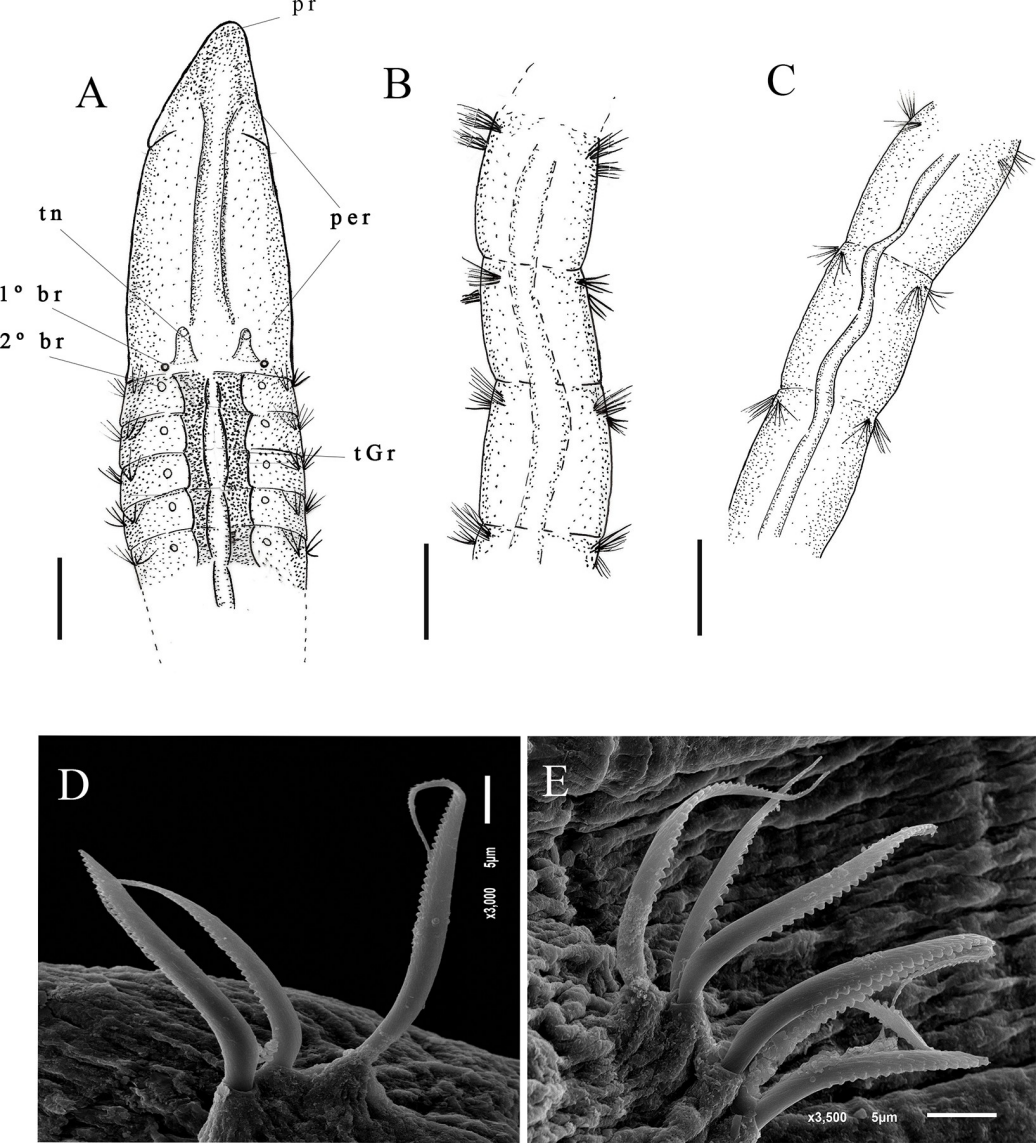
*Kirkegaardia hampsoni*. (A) anterior end, dorsal view; (B) segments of the anterior abdominal region; (C) segments of the posterior abdominal region; (D) notopodial setae in posterior region; (E) neuropodial capillary denticulated. A–C (MNRJP-002966). Scale bars: 100 μm (A–C). Type locality. Atlantic Ocean: Massachusetts (USA) [27]. Habitat. Fine sediments, 30–150 m depth [27]. In the present study, fine sediments, mainly silt/clay, 19–683 m depth. Distribution. Atlantic Ocean: Massachusetts (USA) [27]; Campos Basin (Brazil).

Description. Largest individual with 58 setigers, 2.3 mm long, thoracic region 0.09 mm wide and 0.2 mm high, abdominal region 0.11 mm wide. All specimens incomplete. Prostomium triangular (Fig 2A). Eyes absent, nuchal organs not observed. Peristomium smooth, elongated with 1–2 rings. Peristomium with dorsal crest extending from peristomium to first thoracic setiger (Fig 2A). Dorsal tentacles inserted in posterior margin of peristomium; first pair of branchiae postero-lateral to the tentacles, second pair located in first setiger; branchiae visible up to abdominal region (Fig 2A). Thoracic region narrow with 9–12 setigers (Fig 2A); dorsal parapodia expanded in thoracic region, forming a mid-dorsal groove, dorsal thoracic groove with elevated narrow mid-dorsal ridge. Abdominal region narrow with anterior segments longer than medial abdominal segments (Fig 2B and 2C). Parapodia reduced with poorly developed lobes, barely visible with light microscopy. Thoracic parapodia with 4–5 noto- and neuropodial smooth capillary setae per segment. Abdominal segments with 4–5 noto- and neuropodial smooth capillaries and denticulate setae per segment (Fig 2D and 2E). Denticulate neurosetae present from the setiger 17. Noto- and neurosetae with well-developed denticles along one edge.

Remarks. The specimens from the Campos Basin are similar to the type specimens of *Kirkegaardia hampsoni* Blake, 2016 [22], described for Massachusetts (USA), by having an elongated, smooth peristomium, a dorsal peristomial crest that extends from the final portion prostomium to the first thoracic setiger, and a mid-dorsal channel or thoracic groove; the region closer to the prostomium that has one or two rings. Specimens from the Campos Basin are similar to *K*. *hampsoni* in having the dorsal tentacles inserted in the posterior portion of the peristomium and by having the first pair of branchiae inserted lateral to the dorsal tentacles on the peristomium, and with the second pair of branchiae arising from the first setiger. *K*. *hampsoni* from the Campos Basin also has a narrow ridge in the middle of the dorsal thoracic groove, and the medial abdominal segments are increasingly shorter and wider as in *K*. *hampsoni*. *K*. *hampsoni* specimens from the Campos Basin differ in having between 9–12 thoracic setigers, instead of 10–15 thoracic setigers as described by Blake [27]. The presence of a peristomial ridge *K*. *hampsoni* places the species in the *Kirkegaardia dorsobranchialis-heterochaeta* group as defined by Blake [22], which includes: *K*. *annulosa*, *K*. *cristata*, *K*. *kladara* and *K*. *hampsoni*. The present work represents the record of *K*. *hampsoni* for the South Atlantic Ocean. *K*. *hampsoni*, was found between 19 to 121 m in the Campos Basin whereas Blake [27] records *K*. *hampsoni* from between 30 to 150 m. According to Blake [22] *K*. *hampsoni* was registered locally as *Tharyx* and/or *Monticellina dorsobranchialis* at various locations on the continental shelf of the US Atlantic, from the Gulf of Maine to the mid-Atlantic, the Campos Basin record points out that *K*. *hampsoni* is a species with extensive distribution, but more in-depth studies on the distribution of *Kirkegaardia* species including *K*. *hampsoni* are needed. The samples from the Campos Basin are not complete, and it is not possible to observe the pre-pygidial region and the pygidium.

***Kirkegaardia blakei* sp. nov.** urn:lsid:zoobank.org:act:9DC8FAC8-2445-4FBA-BE7B-8A0235CDE991

Fig 3

Material examined. BRAZIL: Campos Basin–Holotype–-19.69275556°S -39.52138889°W, 48 m, 12/12/10, (MNRJP-002971); Paratypes–-19.91560000°S -39.94467500°W, 32 m, 16/12/10, one ind., (MNRJP-002972); -21.18725000°S -40.09813889°W, 683 m, 04/02/09, one ind., (MNRJP-002973).

Diagnosis. Abdominal region with simple capillaries, gradually replaced by denticulated capillaries. Pre-pygidial abdominal region slightly expanded with 8–10 setigers, with long setae with curved tips and simple denticulated setae.

**Fig 3:**
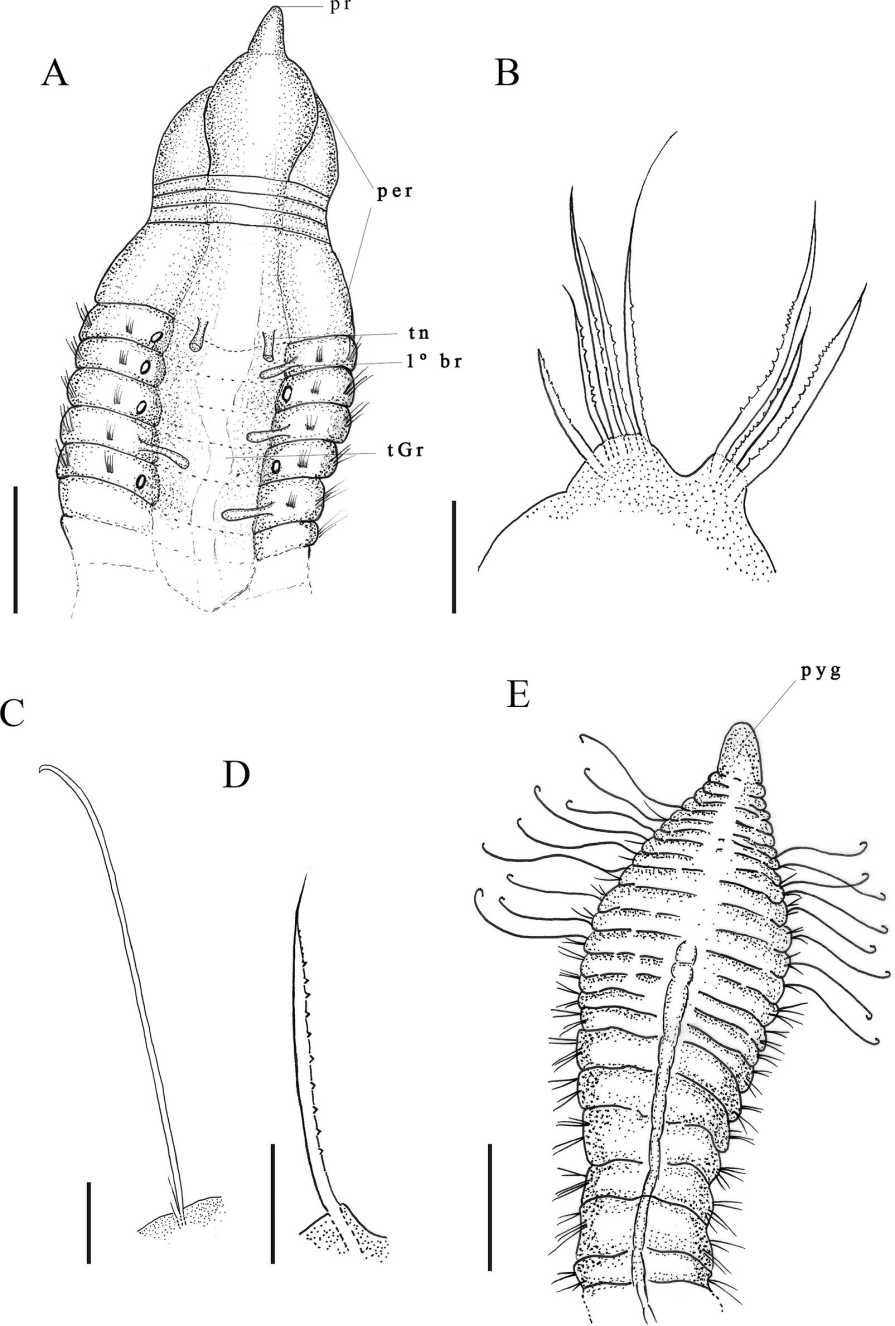
*Kirkegaardia blakei* sp. nov. (A) anterior end, dorsal view; (B) posterior parapodium, anterior view; (C) notopodial seta with long curved tips; (D) neuropodial denticulated capillary; (E) posterior end, dorsal view. A–E, (MNRJP-002971). Scale bars: 100 μm (A and E); 200 μm (B–D). Type locality. Atlantic Ocean: Campos Basin (Brazil). Habitat. Sandy sediments, 38 and 683 m depth. Distribution. Presently known only from Atlantic Ocean: Campos Basin (Brazil). Methyl Green. No pattern, stain not retained.

Description. Complete holotype with 55 setigers, 3 mm long, 0.1 mm wide in thoracic region, and 0.09 mm wide in abdominal region. Prostomium triangular and narrow (Fig 3A); Eyes absent. Peristomium large, with 3–4 narrow rings (Fig 3A). Dorsal tentacles on posterior margin of peristomium (Fig 3A). Thoracic region expanded with 7–8 setigers, thoracic dorsal groove barely visible (Fig 3A). First pair of branchiae inserted dorsolaterally on setiger 1 (Fig 3A), present up to abdominal region. Abdominal setigers narrow. Parapodia between middle abdominal and pre-pygidial segments with slightly elevated lobes (Fig 3B). Thoracic region with setae simple capillaries; abdominal region with 4–5 denticulated noto and neurosetae (Fig 3B) that gradually replace most smooth capillaries; pre-pygidial abdominal region with long setae with curved tips (Fig 3C) and denticulated capillaries (Fig 3D). Pre-pygidial region slightly expanded with 8–10 setigers (Fig 3E). Pygidium slightly expanded, with conical ventral lobe (Fig 3E).

Remarks. *Kirkegaardia blakei* sp. nov. has the peristomial rings with 3–4 very well-marked and concentrated rings at the end of the peristomium. According to Blake [22], these rings are usually smooth, and often difficult to see in optical microscopy, sometimes requiring SEM for verification. *Kirkegaardia blakei* sp. nov. has noto and neurosetae denticulated capillaries in the abdominal region, and unusual long setae modified with a gently curved tip, without denticles or fibrils in the pre-pygidial one. The abdominal region of *K*. *blakei* sp. nov. is similar to *K*. *hampsoni*, however *K*. *hampsoni* has only denticulated setae. For all these characteristics, the species was considered new for science.

Etymology. This species is named in honor of Dr. James Blake for his important works that contributed to the knowledge of polychaetes, including the family Cirratulidae.

***Kirkegaardia brisae* sp. nov.** urn:lsid:zoobank.org:act:F91EA4FC-D190-405B-82B7-675FF65BBDCD

Fig 4

Material examined. BRAZIL: Campos Basin–Holotype–-19.87490556°S -39.81891667°W, 41 m, 16/07/11, (MNRJP-002974); Paratypes–-19.87490556°S -39.81891667°W, 41 m, tree ind., 16/07/11, (MNRJP-002975); -19.69275556°S -39.52159444°W, 48 m, 20 ind., 12/12/10, (MNRJP-002976); -19.87652222°S -39.81822222°W, 35 m, six ind., 16/12/10, (MNRJP-002977); -19.83260556°S -39.87056111°W, 37 m, one ind., 15/12/10, (MNRJP-002978); -19.69027500°S -39.52233889°W, 44 m, 17 ind., 13/07/11, (MNRJP-002979); -19.58430556°S -39.64418333°W, 41 m, one ind., 14/07/11, (MNRJP-002980); -19.95898889°S -39.89250278°W, 46 m, one ind., 17/07/11, (MNRJP-002981); -19.62828611°S -39.59208889°W, 35 m, one ind., 15/07/11, (MNRJP-002982); -19.92933056°S -39.76148611°W, 43 m, one ind., 16/07/11, (MNRJP-002983); -19.83788611°S -39.66978611°W, 48 m, one ind., 15/07/11, (MNRJP-002984); -19.76963611°S -39.58217778°W, 44 m, one ind., 14/07/11, (MNRJP-002985); -19.78693056°S -39.92095000°W, 14 m, seven ind., 16/07/11, (MNRJP-002986); -19.91400833°S -39.94639167°W, 32 m, one ind., 16/07/11, (MNRJP-002987); -19.76538611°S -39.50715000°W, 121 m, one ind., 15/01/12, (MNRJP-002988); -19.76025000°S -39.59519167°W, 352 m, one ind., 28/06/13, (MNRJP-002989).

Diagnosis. Thoracic parapodia elevated above dorsal surface producing a groove between notopodia. Two or three rings on anterior region of peristomium. Pre-pygidial region slightly expanded with pygidium formed by an enlarged ventral lobe.

**Fig 4:**
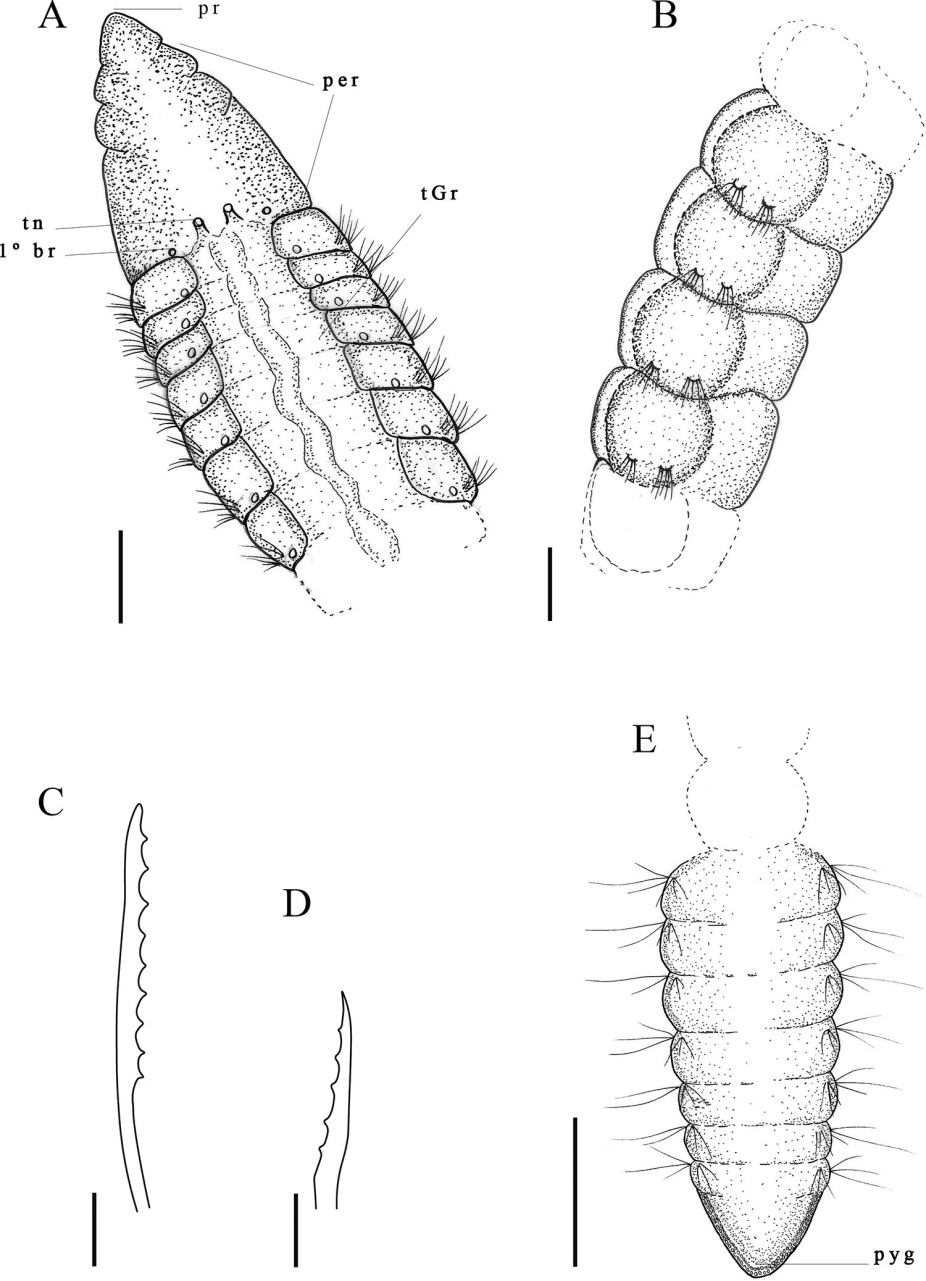
*Kirkegaardia brisae* sp. nov. (A) anterior end, dorsal view; (B) abdominal segments, lateral view; (C) notopodial capillary denticulated; (D) neuropodial capillary denticulated; (E) posterior end, dorsal view. A–E (MNRJP-002974). Scale bars: 100 μm (A B and E); 10 μm (C-D). Type locality. Atlantic Ocean: Campos Basin (Brazil). Habitat. Frequent in sandy sediments, rarer in muddy or sediments with gravels and rhodoliths, 14 to 352 m depth. Distribution. Presently known only from Atlantic Ocean: Campos Basin (Brazil). Methyl Green. No pattern, stain not retained.

Description. Complete holotype with 90 setigers, 2.8 mm long, thoracic region 0.08 mm wide, abdominal region 0.12 mm wide. Prostomium large conical (Fig 4A); eyes absent. Peristomium large with two or three rings visible with optical microscope (Fig 4A). Dorsal tentacles on posterior margin of peristomium (Fig 4A). First pair of branchiae postero-lateral to the tentacles, second pair of branchiae inserted on setiger one dorsal to notosetae (Fig 4A); branchiae absent on abdominal segments. Thoracic region slightly expanded, with 8–10 setigers; dorsal thoracic groove visible in light microscope (Fig 4A). Abdominal segments wider than long (Fig 4B). Parapodia with poorly developed lobes, difficult to see under optical microscope. Thoracic parapodia with 3–7 simple capillaries per segment. Posterior abdominal parapodia with 3–5 noto- and denticulated neurosetae per fascicle (Fig 4C and 4D). Denticulated neurosetae appear first between the abdominal setigers 10 and 24. Pre-pygidial region slightly expanded (Fig 4E). Pygidium formed by an enlarged ventral lobe (Fig 4E).

Remarks. *Kirkegaardia brisae* sp. nov. is similar to *K*. *cryptica* (Blake, 1996) [2]. These species have a conical and wide prostomium, tentacles in the posterior portion of the peristomium, the first pair of branchiae postero-lateral to the tentacles and noto- and neurosetae denticulate. *K*. *brisae* sp. nov. differs from *K*. *cryptica* in that it has three rings with the first two short and narrow and the third longer than the two short ones instead of being smooth and without annulations as in *K*. *cryptica*. *Kirkegaardia brisae* sp. nov. is similar to *K*. *annulosa* (Hartman, 1965) [37] due to the location of peristomial rings right after the prostomium, but *K*. *annulosa* has a peristomial and thoracic crest, while *K*. *brisae* sp. nov. has no crest. *K*. *brisae* sp. nov. is also similar to *K*. *baptisteae* (Blake, 1991) [27] in that they both have an expanded pre-pygidial region, but the latter differs in having a pygidium formed by a narrow terminal lobe. In contrast, *K*. *brisae* sp. nov. has a terminal lobe expanded distally. For these characteristics, the species was considered as new to science.

Etymology. This species is named in honor of Mrs. Suzanne Marie Thérèse Bris in recognition of her encouragement to young scientists in the study of polychaetes.

***Kirkegaardia goytaca* sp. nov.** urn:lsid:zoobank.org:act:0C21AF49-C7AD-4686-8AD6-CE74CEB54ABA

Fig 5

Material examined. BRAZIL: Campos Basin–Holotype–-19.95913611°S -39.89185833°W, 43 m, 16/12/10, (MNRJP-002990); Paratypes–-19.59332222°S -39.68905833°W, 21 m, one ind., 16/07/13, (MNRJP-002991).

Diagnosis. Peristomium and thoracic region with dorsal crest joining with and continuing with the peristomial crest. Noto- and neurosetae denticulated.

**Fig 5:**
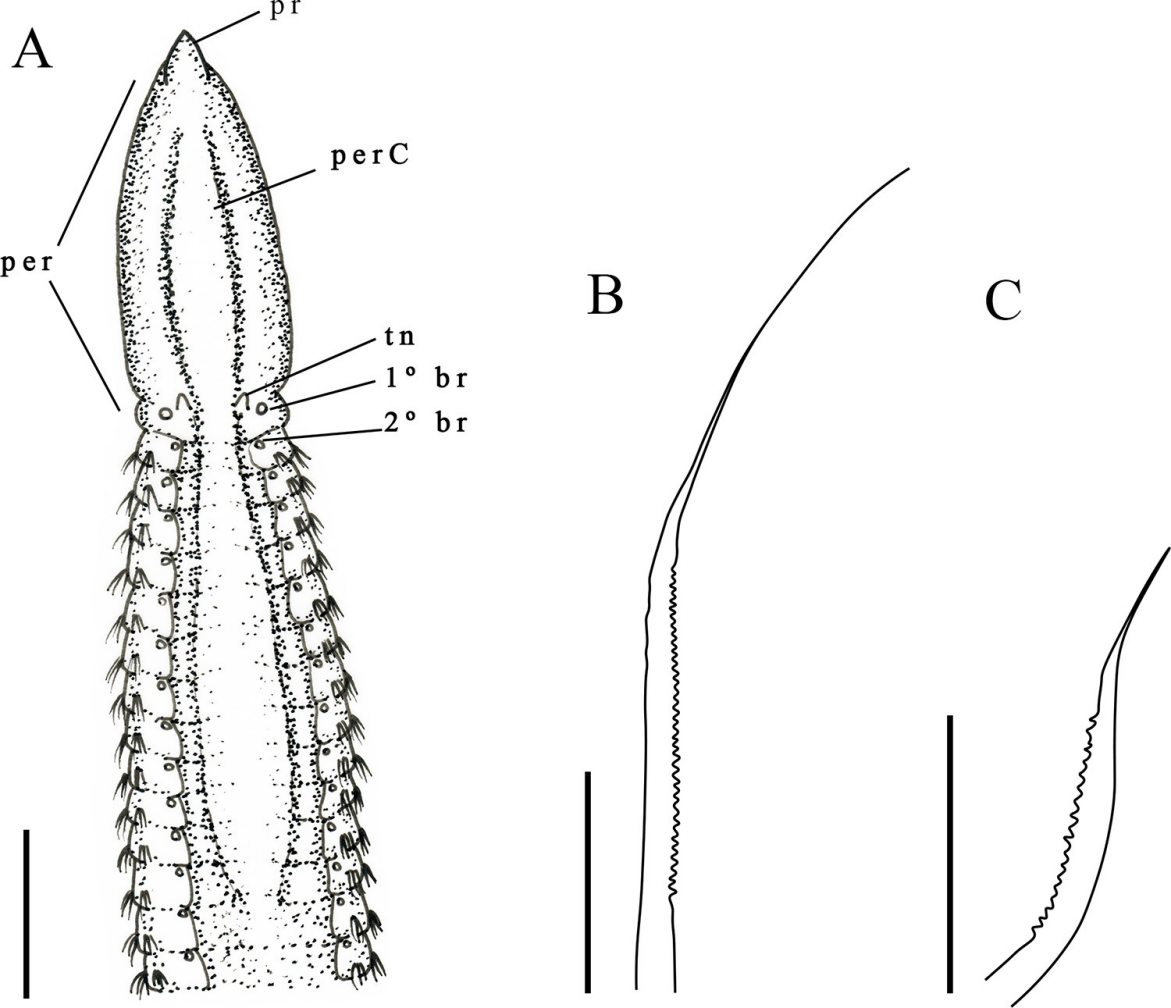
*Kirkegaardia goytaca* sp. nov. (A) anterior end, dorsal view; (B) notopodial capillary denticulated; (C) neuropodial capillary denticulated. A–C (MNRJP-002990). Scale bars; 200 μm (A-C). Type locality. Atlantic Ocean: Campos Basin (Brazil). Habitat. Fine sediments (clay) and sandy sediments organically enriched, 21 and 43 m depth. Distribution. Presently known only from Atlantic Ocean: Campos Basin (Brazil). Methyl Green. No pattern, stain not retained.

Description. Holotype with 76 setigers, body 4.6 mm long, 0.12 mm wide in thoracic region, and 0.13 mm wide in abdominal region. All specimens incomplete. Prostomium short conical (Fig 5A); Eyes absent. Peristomium elongate, with a single short posterior ring; with a prominent dorsal crest along most of length, merging with thoracic crest (Fig 5A). Dorsal tentacles on short peristomial ring (Fig 5A). First pair of postero-lateral branchiae to the tentacles; second pair of branchiae in setiger 1 dorsal to notosetae (Fig 5A), continuing segmentally to middle of abdominal region. Thoracic region narrowing after about 14–16 setigers, with a thoracic crest ending. After end of thoracic crest, thoracic region with a narrow groove, visible in light microscope (Fig 5A). Abdominal segments wider than long. Parapodia with poorly developed lobes. Thoracic parapodia with 5–6 capillary noto- and neurosetae per segment. Posterior abdominal parapodia with 4–5 denticulated notosetae (Fig 5B) and six or seven denticulated neurosetae per segment (Fig 5C). Denticulated neurosetae appear from abdominal setigers 30 to 38. Abdominal neurosetae smaller than notosetae in first abdominal segments, noto- and neurosetae of same size in middle segments of abdominal region. Pre-pygidial segments and pygidium absent.

Remarks. *Kirkegaardia goytaca* sp. nov. is similar to *K*. *carinata* Blake, 2016 [22] in that both have a narrow body, abdominal segments wider than long, thoracic crest, tentacles in the posterior portion of the peristomium, and first pair of postero-lateral branchiae to the tentacles between the peristomium and the first setiger. However, *K*. *carinata* presents the first pair of branchiae inserted in the groove that separates the peristomium from the first setiger, while *K*. *goytaca* sp. nov. has the first pair of branchiae inserted in the peristomial ring. In addition, *K*. *goytaca* sp. nov. has a peristomial crest, while *K*. *carinata* only thoracic crest. The presence of a peristomial crest throughout the peristomium brings *K*. *goytaca* sp. nov., *K*. *jongo* sp. nov. and *K*. *papaveroi* sp. nov. to a group of four species, *Kirkegaardia dorsobranchialis-heterochaeta*. *Kirkegaardia goytaca* sp. nov. differs from *K*. *cristata* in having only a single ring at the end of the peristomium, while *K*. *cristata* has three well-marked peristomial rings. *Kirkegaardia goytaca* sp. nov. differs from *K*. *annulosa* in having the thoracic region very narrow, while *K*. *annulosa* has a thoracic region expanded. *Kirkegaardia goytaca* sp. nov. also differs from *K*. *kladara* in having the first pair of branchiae inserted in the final portion of the peristomium, while in *K*. *kladara* the first pair of branchiae occurs from the first thoracic setiger. For all these characteristics, the species was considered as new to science.

Etymology. This name is in honor of the extinct local ethnic group uetaká, also known as Goytacá. These native people lived along lowlands from the northern Macaé River to Espírito Santo State, a coastal region that comprises a large part of the sampling sites where this and the other *Kirkegaardia* species of the present study were collected.

***Kirkegaardia helenae* sp. nov.** urn:lsid:zoobank.org:act:A8469F0C-EC3F-4C39-BCE7-9F7252F9B84E

Fig 6

Material examined. BRAZIL: Campos Basin–Holotype–-19.60138889°S -39.17581389°W, 145 m, 21/04/12, (MNRJP-002992); Paratypes–-19.60138889°S -39.17581389°W, 145 m, one ind., 21/04/12, (MNRJP-002993); -20.57621389°S -40.34743611°W, 21 m, one ind., 12/07/13, (MNRJP-002994); -19.60850000°S -39.17205278°W, 349 m, two ind., 26/06/12, (MNRJP-002995); -19.78693056°S -39.92095000°W, 14 m, one ind., 16/07/11, (MNRJP-002996).

Diagnosis. Peristomium large, with 3–4 rings, first pair of branchiae postero-lateral to the tentacles in setiger 1. Noto and neurosetae denticulate in abdominal region. Pre-pygidial abdominal region prominently expanded.

**Fig 6:**
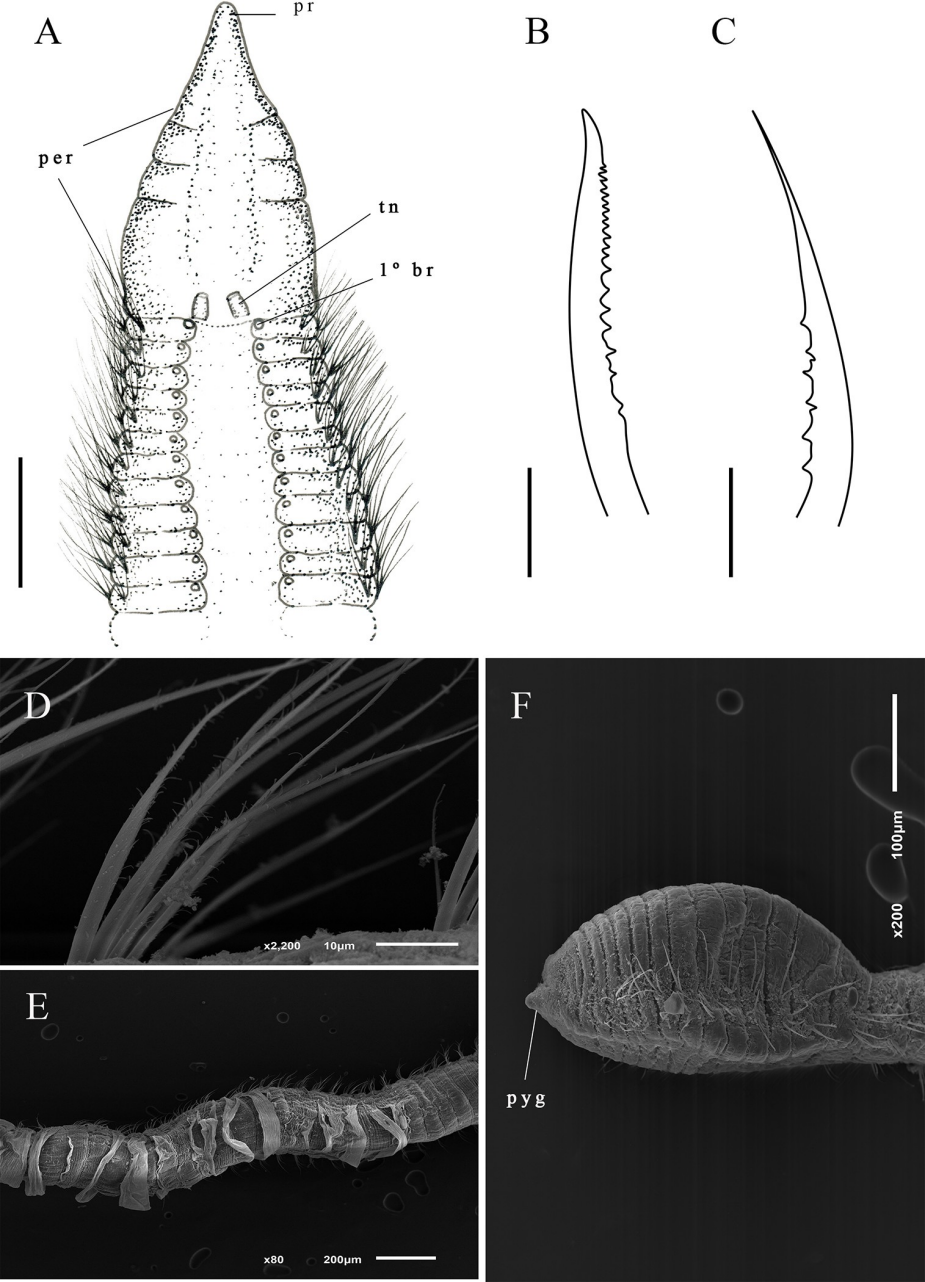
*Kirkegaardia helenae* sp. nov. (A) anterior end, dorsal view; (B) neuropodial capillary denticulated; (C) notopodial capillary denticulated; (D) capillaries in thoracic setigers with fibrils; (E) abdominal segments, dorsal view; (F) posterior end, lateral view. A–C (MNRJP-002992). Scale bars: 100 μm (A) 200 μm (B and C). Type locality. Atlantic Ocean: Campos Basin (Brazil). Habitat. Thin silt and clay sediments, 14 to 349 m depth. Distribution. Presently known only from Atlantic Ocean: Campos Basin (Brazil). Methyl Green. Parapodia with stain concentrated on posterior borders of noto- and neuropodia along entire body.

Description. Complete holotype with 80 setigers, body 2.2 mm long, 0.11 mm wide in across thoracic region, and 0.13 mm wide in abdominal region. Prostomium conical, narrow (Fig 6A); eyes absent. Peristomium large, with 3–4 rings visible under the light microscope (Fig 6A). Dorsal tentacles on mid-posterior margin of peristomium (Fig 6A); first pair of branchiae arises postero-lateral to the tentacles on setiger 1 medial to notosetae; subsequent branchiae in same location on following segments (Fig 6A); branchiae present up to the pre-pygidial abdominal region. Thoracic region expanded with 12–18 setigers, dorsal groove absent. Parapodia with poorly developed lobes, difficult to see with optical microscopy. Thoracic parapodia with 5–7 neuro- and notosetae capillary with fringe of stiff, sparsely occurring fibrils along shaft (Fig 6D). Abdominal segments short, wider than long (Fig 6E). Posterior abdominal parapodia with 4–8 denticulated notosetae and 7–10 denticulated neurosetae per segment (Fig 6B and 6C). Denticulated neurosetae appear between the abdominal setigers 27 and 31. Pre-pygidial segments greatly expanded, rounded dorsally, and flattened ventrally, with lateral parapodia (Fig 6F). Pygidium formed by a ventral conical lobe (Fig 6F).

Remarks. *Kirkegaardia helenae* sp. nov. belongs to the *Kirkegaardia baptisteae*-*tesselata* group, due to their similar morphology, species in this group have elevated thoracic parapodia, and a mid-dorsal channel is not produced, although a dorsal crest sometimes develops. *Kirkegaardia helenae* sp. nov. is similar to *K*. *zafirae* sp. nov. and *K*. *baptisteae* in that it presents denticulated noto and neurosetae, although it differs from both species in that its first pair of branchiae appear in the first setigerous and not in the posterior region of the peristomium. *Kirkegaardia helenae* sp. nov. also differs from *K*. *zafirae* sp. nov. in that it does not have a ventral sulcus in the pre-pygidial region. The shape of the pre-pygidial region of *K*. *helenae* sp. nov. is similar to that of *K*. *lueldredgei*, but it has abdominal segments that are wider than long, unlike *K*. *lueldredgei*, which has abdominal segments that are longer than wide. These species also differ in the number of neurosetae in the posterior abdominal region, two or three setae in *K*. *lueldredgei*, and 7–10 in *K*. *helenae* sp. nov. The disposition of the tentacles and first pair of branchiae of *K*. *helenae* sp. nov. is similar to *K*. *setosa* (Dean & Blake, 2009) [38] but this species has only denticulate neurosetae and *K*. *helenae* sp. nov. has noto and neurosetae denticulate in its abdominal region. For these characteristics, the species was considered as new to science.

Etymology. This species is named in honor of Dr. Helena Passeri Lavrado, for her important contribution to Brazilian marine biology.

***Kirkegaardia jongo* sp. nov.** urn:lsid:zoobank.org:act:5516ACDD-13DE-482E-88B8-8A1BF6C949E2

Fig 7

Material examined. BRAZIL: Campos Basin–Holotype–-20.06893889°S -38.52425556°W, 1288 m, 21/12/11, (MNRJP-002997); Paratypes–-20.60056389°S -39.85982500°W, 991 m, one ind., 08/01/2012, (MNRJP-002998); -19.82691667°S -39.59519167°W, 352 m, one ind., 28/06/13, (MNRJP-002999).

Diagnosis. Presence of a peristomial crest along the peristomium. Peristomium with two or three annulations. Thoracic region without visible crest. Noto and neurosetae denticulated. Presence of a small segment, demarcating the separation of the pre-pygidial region from the other abdominal segments.

**Fig 7:**
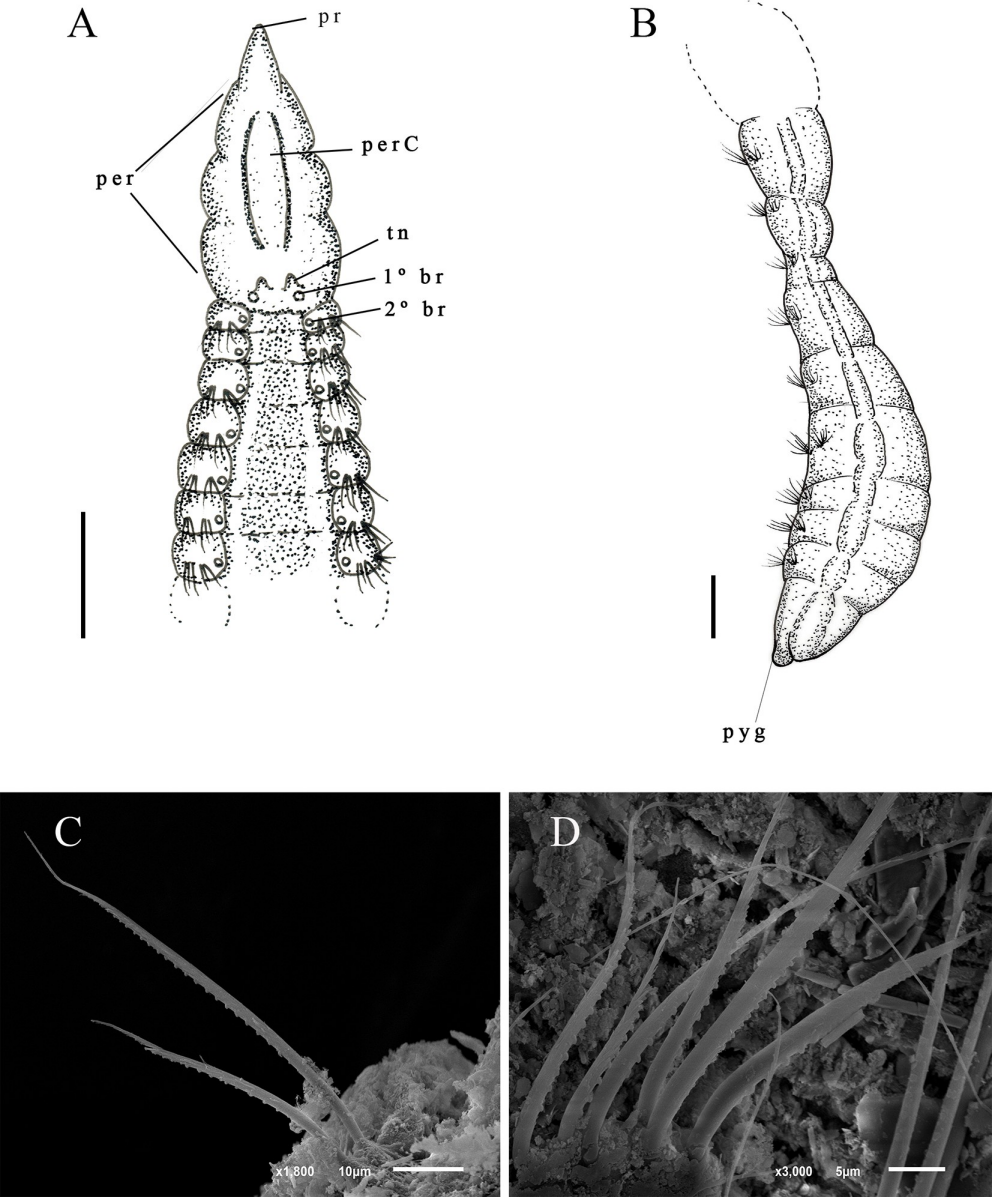
*Kirkegaardia jongo* sp. nov. (A) anterior end, dorsal view; (B) posterior end, lateral view; (C) denticulated notopodial capillary; (D) denticulated neuropodial capillary. A–B (MNRJP-002997). Scale bars: 100 μm (A and B). Type locality. Atlantic Ocean: Campos Basin (Brazil). Habitat. Fine sediments composed of silt and clay, or sand, depth between 352 to 1288m. Distribution. Presently known only from Atlantic Ocean: Campos Basin (Brazil). Methyl Green. No pattern, stain not retained.

Description. Complete holotype with 58 setigers, 2.4 mm long, 0.2 mm wide in thoracic region, and 0.07 mm wide in abdominal region. Prostomium narrow, triangular (Fig 7A). Eyes absent. Peristomium with 2–3 rings and dorsal crest (Fig 7A). Dorsal tentacles on posterior margin of peristomium (Fig 7A). First pair of branchiae postero-lateral to the tentacles on the posterior most border of peristomium; second pair of branchiae in setiger 1 dorsal to notosetae (Fig 7A); branchiae continuing to anterior abdominal segments. Thoracic region with nine setigers, slightly expanded; dorsal groove barely visible with optical microscope. Abdominal segments wider than long. Parapodia with poorly developed lobes, difficult to see with optical microscopy. Thoracic parapodia with 4–5 capillary noto- and neurosetae per segment. Posterior abdominal parapodia with 4–6 denticulate noto- and neurosetae (Fig 7C and 7D) per segment. Denticulate neurosetae from abdominal setiger 17. Pre-pygidial segments well-defined, marked by the presence of a small narrow segment, separating pre-pygidial region from other abdominal segments (Fig 7B). Pygidium small, rounded ventral lobe (Fig 7B).

Remarks. The presence of a peristomial crest brings *K*. *jongo* sp. nov. as well as *K*. *papaveroi* sp. nov. and *K*. *goytaca* sp. nov. to the group of four species considered to be from deep sea: *K*. *annulosa*, *K*. *cristata*, *K*. *kladara* and *K*. *hampsoni*. Among these species, *K*. *hampsoni* and *K*. *jongo* sp. nov. do not have a visible crest in the thoracic region. *K*. *annulosa* differs from *K*. *jongo* sp. nov. by having hooks in addition to modified setae, and pre-pygidial segments ventrally flattened culminating in a conical pygidial lobe. *Kirkegaardia jongo* sp. nov. has no hooks, and the pre-pygidial segments are fully expanded, culminating in a small rounded pygidial lobe. *Kirkegaardia kladara* differs from *K*. *jongo* sp. nov. by the presence of a thoracic crest and with the first pair of branchiae on setiger 1, whereas in *K*. *jongo* sp. nov. the first pair of branchiae are on the posterior margin of the peristomium. *Kirkegaardia cristata*, differs from *K*. *jongo* sp. nov. in having an extremely long prostomium and moniliform segments in the abdominal region while *K*. *jongo* sp. nov. has a triangular prostomium, and abdominal segments wider than long. *K*. *cristata* also has a thoracic ridge, and *K*. *jongo* sp. nov. does not have a thoracic crest. *Kirkegaardia hampsoni* differs from *K*. *jongo* sp. nov. in having a peristomium without rings, while *K*. *jongo* sp. nov. has two or three annulations in the peristomium, and by the number of abdominal setae per fascicle, 8–12 in *K*. *hampsoni*, and 4–6 in *K*. *jongo* sp. nov. For all these differences, the species was considered as new to science.

Etymology. This species is named after the quilombolan dance known as “jongo”, as a tribute to the African legacy left in the southeastern Rio de Janeiro State, region that comprises a large part of the sampling points where *Kirkegaardia jongo* has been collected.

***Kirkegaardia medusa* sp. nov.** urn:lsid:zoobank.org:act:4A38559A-3F95-419F-B99A-64E893478AB5

Fig 8

Material examined. BRAZIL: Campos Basin–Holotype–-20.69275833°S -39.58946111°W, 41 m, 17/07/13, (MNRJP-003000); Paratypes–-19.60138889°S -39.17555556°W, 148 m, four ind., 24/01/12, (MNRJP-003001); -19.60728889°S -39.17148611°W, 352 m, one ind., 14/12/11, (MNRJP-003002).

Diagnosis. Peristomium with one ring. First pair of branchiae on setiger one. Notosetae with fibrils in abdominal region. Only neurosetae denticulated, present from abdominal setigers 15 or 16.

**Fig 8:**
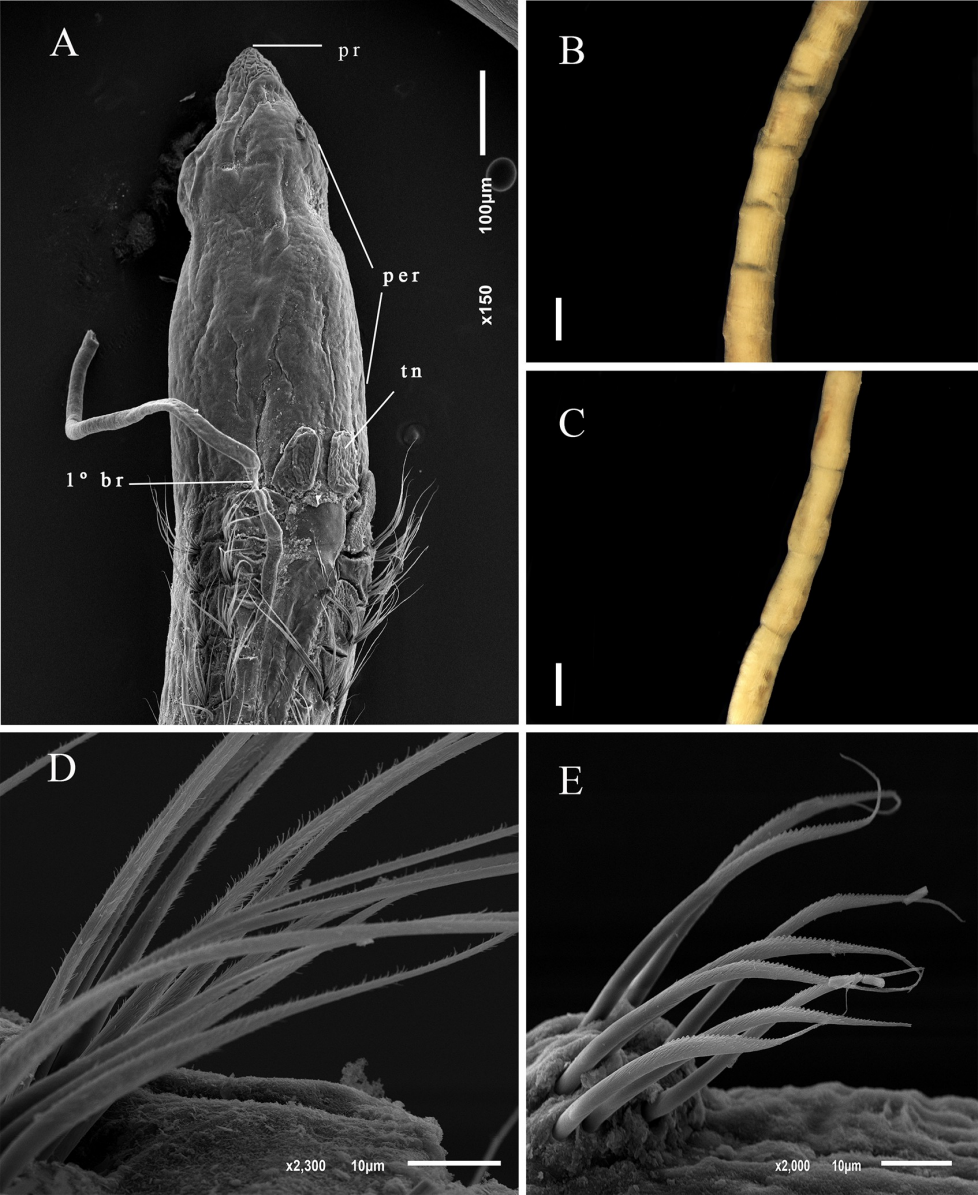
*Kirkegaardia medusa* sp. nov. (A) anterior end, dorsal view; (B) abdominal segments, lateral view; (C) posterior abdominal segments, lateral view; (D) notopodial capillary with fibrils in abdominal region (E) neuropodial capillary denticulated. B–C (MNRJP-003000). Scale bars: 2 mm (B and C). Type locality. Atlantic Ocean: Campos Basin (Brazil). Habitat. Fine sediments composed of silt and clay, between 41 to 352 m deep. Distribution. Presently known only from Atlantic Ocean: Campos Basin (Brazil). Methyl Green. No pattern, but prominent staining on the thoracic region.

Description. All specimens incomplete, holotype with 43 setigers, 9.0 mm long, 0.12 mm wide in the thoracic region, and 0.10 mm wide in abdominal region. Prostomium long, conical (Fig 8A). Eyes absent. Peristomium with one ring, without dorsal crest (Fig 8A). Dorsal tentacles on posterior margin of peristomium (Fig 8A). First pair of branchiae postero-lateral to the tentacles in setiger 1, second pair of branchiae in setiger 2; branchiae present only in thoracic region. Thoracic region narrow, with 7–10 setigers, without dorsal groove (Fig 8A). Abdominal segments longer than wide (Fig 8B and 8C); first abdominal segments shorter than median abdominal segments (Fig 8B). Parapodia with poorly developed lobes. Thoracic parapodia with four or five capillary noto- and neurosetae per segment. Posterior abdominal parapodia with four or five capillary notosetae simple with numerous thin fibrils (Fig 8D), and 4–8 denticulate neurosetae per fascicle (Fig 8E). Denticulate neurosetae from abdominal setigers 15 or 16. Pre-pygidial segments and pygidium not observed.

Remarks. *Kirkegaardia medusa* sp. nov. is similar to *K*. *baptisteae* (Blake, 1991) [27], *K*. *dutchae* Blake, 2016 [22], *K*. *neotesselata* Blake, 2016 [22] and *K*. *serratiseta* (Banse & Hobson, 1968) [25] in only having denticulate neurosetae. *Kirkegaardia baptisteae* has a nuchal organ lateral to the prostomium and first pair of branchiae anterior to the tentacles. In contrast, the first pair of branchiae from *K*. *medusa* sp. nov. is postero-lateral to the tentacles. They also differ in that, *K*. *baptisteae* has abdominal segments that are wider than long, while *K*. *medusa* sp. nov. has abdominal segments that are longer than wide, up to the median abdominal region. *Kirkegaardia dutchae* differs from *K*. *medusa* sp. nov. by the presence of a peristomial crest and the lateral position of the 1st pair of branchiae to the tentacles. *Kirkegaardia neotesselata* differs has an elevated peristomial crest that merges with the thoracic crest, while *K*. *medusa* sp. nov. has no crest. *K*. *serratisseta* has a thoracic region with about 40 segments, while *K*. *medusa* sp. nov. only has 7 to 10 abdominal segments. For all these characteristics, the species was considered as new to science.

Etymology. This species name refers to the Greek myth of Medusa. Medusa is often seen as evil but was convicted of a crime committed by the God Poseidon, who is more powerful and less vulnerable. This species name represents the fight against misogyny, which is suitable for a study performed by female researchers.

***Kirkegaardia nupem* sp. nov.** urn:lsid:zoobank.org:act:61CADA34-741B-4321-B8E0-1A170BE2937C

Fig 9

Material examined. BRAZIL: Campos Basin–Holotype–-19.60158333°S -39.17581389°W, 1010 m, 21/04/12, (MNRJP-003003); Paratypes–-19.60158333°S -39.17581389°W, 148 m, one ind., 24/01/12, (MNRJP-003004); -19.95888889°S -39.89185833°W, 43 m, one ind., 16/12/10, (MNRJP-003005); -21.39441667°S -40.26077778°W, 88 m, one ind., 21/07/09, (MNRJP-003006); -21.38483333°S -40.25302778°W, 140 m, one ind., 21/07/09, (MNRJP-003007).

Diagnosis. Peristomium smooth, without rings, with peristomial crest. First pair of branchiae postero-lateral to the tentacles first setiger; branchiae present up to pre-pygidial segments. Noto and neurosetae denticulated. Pre-pygidial segments not expanded.

**Fig 9:**
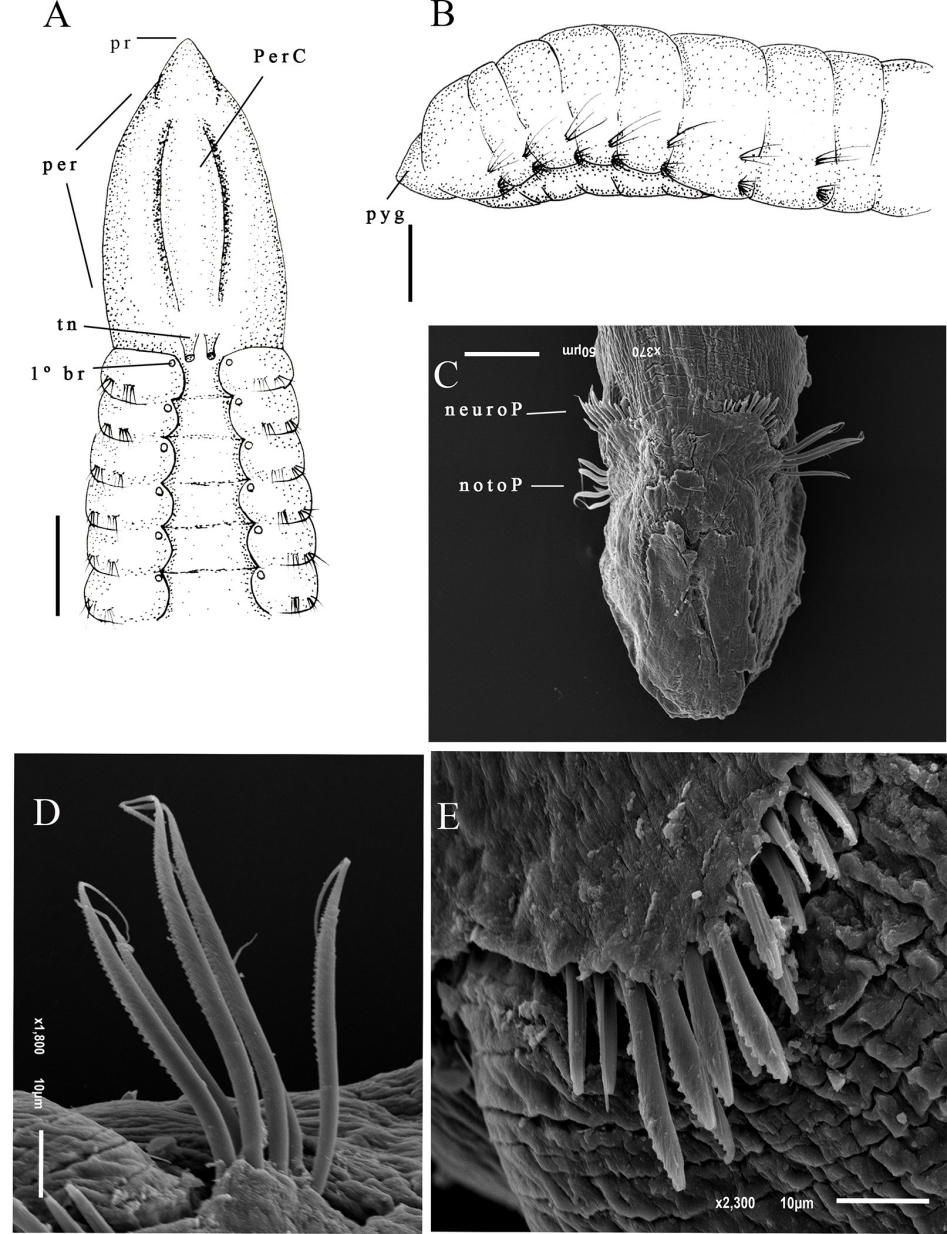
*Kirkegaardia nupem* sp. nov. (A) anterior end, dorsal view; (B) posterior end, lateral view; (C) posterior end with tube, ventral view; (D) notopodial setae in posterior region; (E) neuropodial setae in posterior region. A and B (MNRJP-003003). Scale bars: 100 μm (A and B). Type locality. Atlantic Ocean: Campos Basin (Brazil). Habitat. Sandy sediments with or without biodebris, 46 to 1010 m depth. Distribution. Presently known only from Atlantic Ocean: Campos Basin (Brazil). Methyl Green. No pattern, stain not retained.

Description. Complete holotype with 80 setigers, 8.0 mm long, 0.10 mm wide in the thoracic region, and 0.12 mm wide in abdominal region. Prostomium short conical (Fig 9A); eyes absent. Peristomium smooth, without rings, with peristomial crest along entire length (Fig 9A). Dorsal tentacles medial, closely spaced on posterior border of peristomium (Fig 9A). First pair of branchiae postero-lateral to the tentacles and dorsal to notosetae on setiger 1; second pair of branchiae in same location on following setigers (Fig 9A); branchiae present up to pre-pygidial segments. Thoracic region narrow with 9–14 setigers, without dorsal groove (Fig 9A). Abdominal segments wider than long (Fig 9B) but longer than wide in some specimens. Parapodia with poorly developed lobes, difficult to see with optical microscopy. Thoracic parapodia with 4–6 noto- and neurosetae capillary per segment. Posterior abdominal parapodia with 5–7 denticulated capillary notosetae with long filamentous tips (Fig 9C and 9D) and 14–16 heavy spinous denticulated neurosetae per segment (Fig 9C–9E). Denticulated neurosetae first appearing between abdominal setigers 27 and 31. Abdominal neurosetae larger than those in pre-pygidial segments. Pre-pygidial segments not expanded (Fig 9B). Pygidium with a ventral lobe (Fig 9B).

Remarks. *Kirkegaardia nupem* sp. nov. is similar to *K*. *carrikeri* (Dean & Blake, 2009) [38] by the conical shape of the prostomium, presence of the peristomial crest, positioning of the dorsal tentacles in the posterior portion of the peristomium, by the first pair of branchiae from setiger 1, and by the presence of a shallow ventral groove in the pre-pygidial region. *Kirkegaardia nupem* sp. nov. differs from *K*. *carrikeri* in that it does not have annulations in the peristomium. The number of noto- and neurosetae in the abdominal region differs between species; *K*. *carrikeri* has from 2 to 4 neurosetae in the posterior abdominal region, whereas *K*. *nupem* sp. nov. has from 14 to 16 neurosetae in the posterior abdominal segments. *Kirkegaardia carrikeri* has only denticulated neurosetae throughout the abdominal region, while *K*. *nupem* sp. nov. has denticulated noto- and neurosetae in the abdominal region. Pre-pygidial segments of *K*. *nupem* sp. nov. resembles *K*. *cryptica* (Blake, 1996) [2] due to the presence of ventral groove, but *K*. *cryptica* has the first pair of branchiae present postero-lateral to the tentacles on the peristomium, while *K*. *nupem* sp. nov. has the first pair of branchiae on setiger 1. For all these characteristics, the species was considered as new to science.

Etymology. The epithet is a reference to the Institute of Biodiversity and Sustainability (NUPEM / UFRJ) acronym, where the beginning of this taxonomic work was developed. Institution recognized for its quality in undergraduate and graduate education, and in the production of scientific knowledge.

***Kirkegaardia papaveroi* sp. nov.** urn:lsid:zoobank.org:act:A21CC609-BAFF-4B0E-AC2F-9A2B5C2F2101

Fig 10

Material examined. BRAZIL: Campos Basin–Holotype–-19.78693056°S -39.92095000°W, 14 m, 16/07/2011, one ind., (MNRJP-003008);–Paratypes–-19.79245278°S -39.72085556°W, 34 m, 15/08/2010, one ind., (MNRJP-003009); -19.87652222°S -39.81823056°W, 42 m, 16/12/2010, one ind., (MNRJP-003010); -19.70894722°S -39.64926667°W, 35 m, 14/12/2010, one ind., (MNRJP-003011); -19.92907222°S -39.76075000°W, 46 m, 16/12/2010, one ind., (MNRJP-003012); -19.74557222°S -39.77555833°W, 29 m, 15/07/2011, two ind., (MNRJP-003013); -19.87490556°S -39.81891667°W, 41 m, 16/07/2011, one ind., (MNRJP-003014); -19.69027500°S -39.52233889°W, 44 m, 13/07/2011, 15 ind., (MNRJP-003015); -19.60143611°S -39.17581389°W, 145 m, 21/04/2012, one ind., (MNRJP-003016).

Diagnosis. Peristomium elongated, with two lateral grooves producing rings. Peristomial dorsal crest present. Neurosetae with denticles; with serrated edge of fibrils along shaft from about 20 abdominal setigers. Pre-pygidial region expanded.

**Fig 10:**
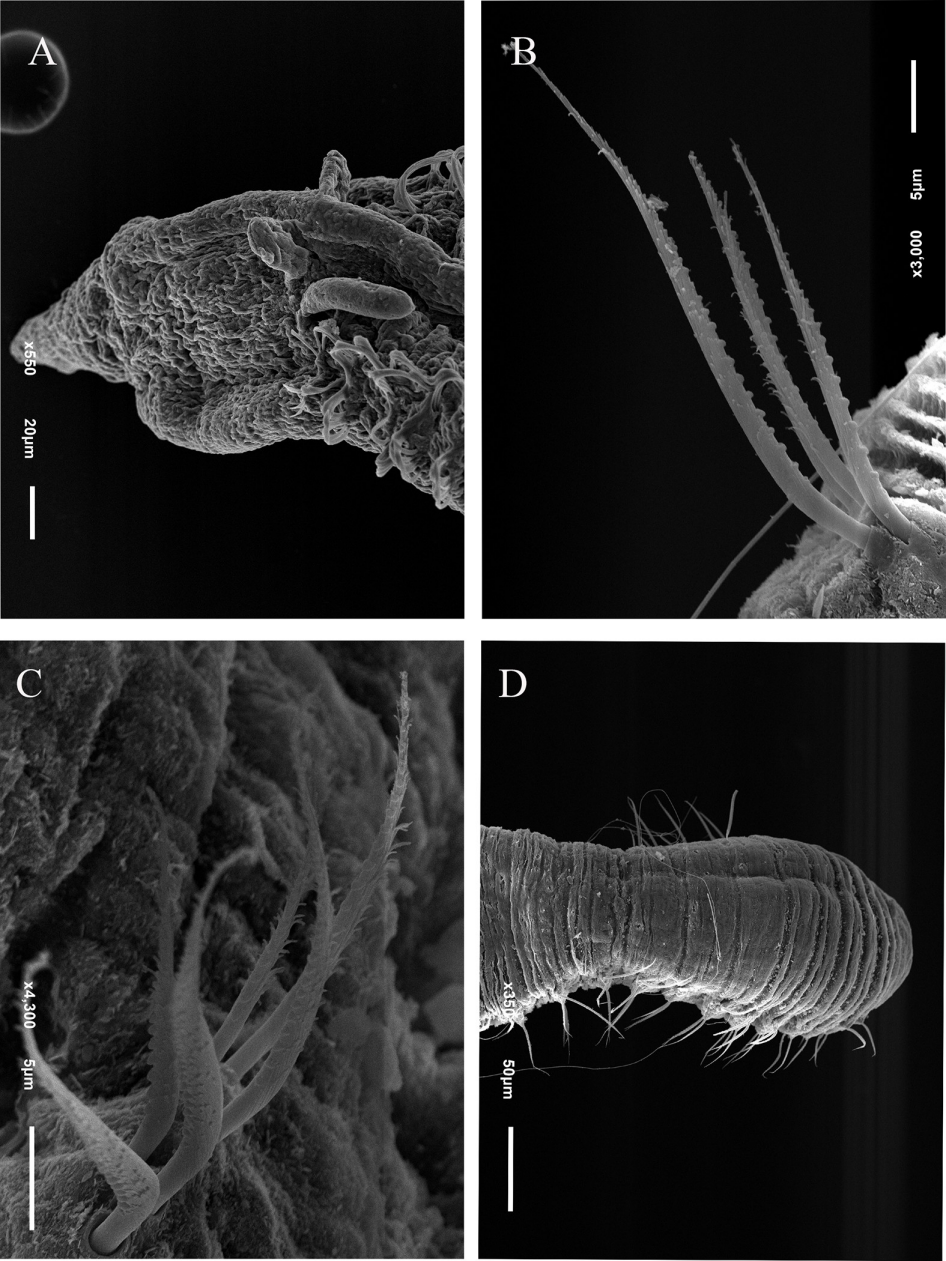
*Kirkegaardia papaveroi* sp. nov. (A) Anterior end, lateral view; (B) notopodial capillary denticulated; (C) neuropodial capillary denticulated; (D) posterior end, dorsal view.

**Fig 11:**
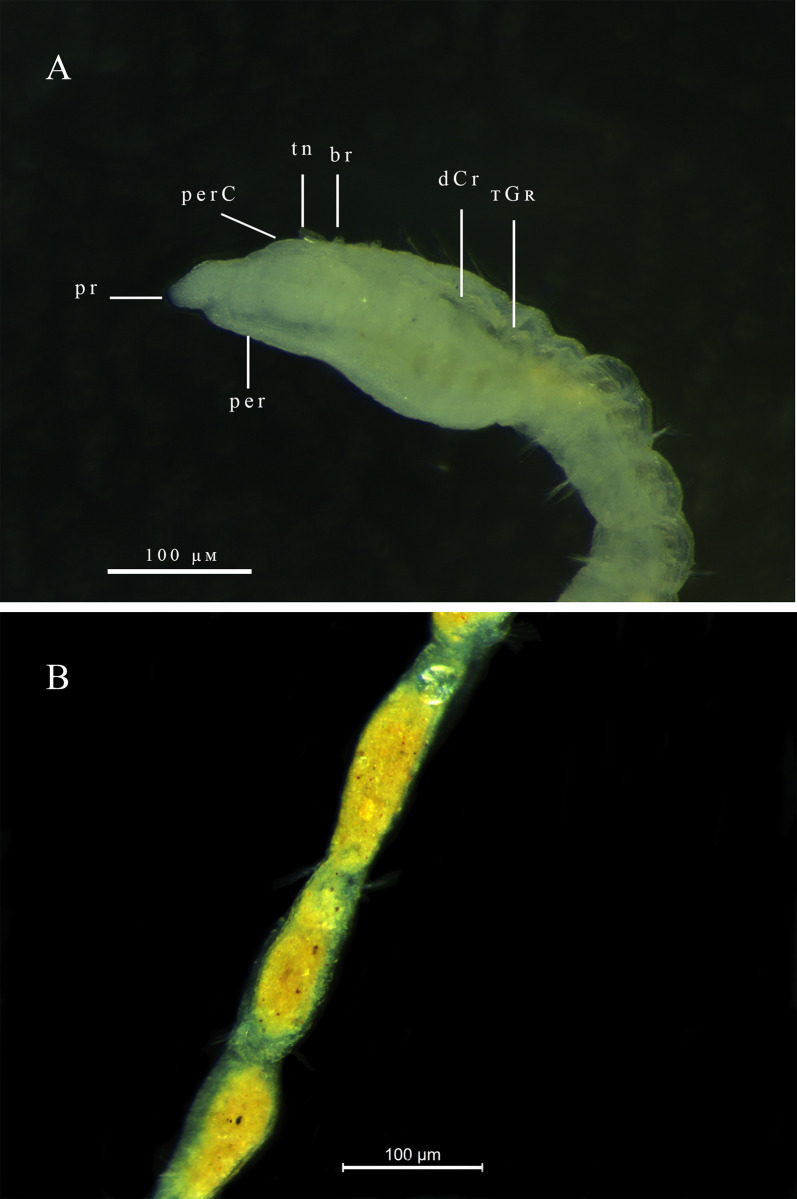
*Kirkegaardia papaveroi* sp. nov. (A) Anterior end, lateral view; (B) abdominal segments, dorsal view. A and B (MNRJP-003008). Type locality. Atlantic Ocean: Campos Basin (Brazil). Habitat. Sandy sediments, 14 and 145 m depth. Distribution. Presently known only from Atlantic Ocean: Campos Basin (Brazil). Methyl Green. No pattern, stain not retained.

Description. Holotype with 63 setigers, 2.4 mm long, thoracic region 0.09 mm wide, 0.1 mm high, abdominal region 0.06 mm wide. Prostomium short, triangular, bluntly rounded on anterior margin (Fig 10A). Eyes absent. Peristomium elongated, with one lateral groove producing two rings, peristomial dorsal crest present (Fig 10A). Dorsal tentacles on posterior margin of peristomium. First pair of branchiae posterolateral to dorsal tentacles at boundary with setiger 1 (Fig 10A), branchiae rarely present in abdominal segments. Thoracic setigers about as wide as long for first nine or ten setigers, with parapodia shifted dorsally and elevated above mid-dorsal surface, forming distinct mid-dorsal groove on thoracic segments (Fig 11A), thoracic crest present (Figs 10A and 11A). Abdominal segments becoming longer and narrower and with parapodia located laterally (Fig 11B). Thoracic parapodia with smooth capillaries numbering about 4–6 in notopodia and neuropodia. Anterior abdominal segments with smooth capillaries in noto- and neuropodia gradually replaced by denticulate capillaries, fascicles reduced to 3–5 setae in abdominal setigers. Noto- and neurosetae heavily serrated or denticulated (Fig 10B and 10C). Neurosetae with denticles and serrated edge of fibrils along shaft from about 20 abdominal setigers (Fig 10C). Pre-pygidial region expanded with dorsal groove (Fig 10D). Pygidium formed by a simple ventral lobe (Fig 10D).

Remarks. The presence of a peristomial crest brings *Kirkegaardia papaveroi* sp. nov. as well as *Kirkegaardia jongo* sp. nov., and *Kirkegaardia goytaca* sp. nov. into the group of species termed *Kirkegaardia dorsobranchialis-heterochaeta* by Blake [27] which includes the species: *K*. *annulosa*, *K*. *cristata K*. *kladara* and *K*. *hampsoni*. *K*. *annulosa* differs from *K*. *papaveroi* sp. nov. by having unusual spike like serrated neurosetae in addition to denticulated capillaries. Among these species, *K*. *hampsoni* and *K*. *jongo* sp. nov. differs from *K*. *papaveroi* sp. nov. by lacking the thoracic dorsal crest. *Kirkegaardia papaveroi* sp. nov. it differs from *K*. *cristata* in that it does not have a prominent ventral sulcus in the first abdominal setigers, in that it has 0–2 distinct rings in the peristomium instead of three. *Kirkegaardia papaveroi* sp. nov. differs from *K*. *kladara* by the branchiae arising lateral to the dorsal tentacles on the peristomium instead of setiger 1. *K*. *goytaca* sp. nov. differs from *K*. *papaveroi* sp. nov. by having the thoracic region extremely narrow. For all these differences, *K*. *papaveroi* was considered as new to science.

Etymology. This species is named in honor of Dr. Nelson Papavero for his important contribution to zoology in Brazil.

***Kirkegaardia zafirae* sp. nov.** urn:lsid:zoobank.org:act:1D92DB03-61C4-4E0B-BA5A-8245AA04BD87

Fig 12

Material examined. BRAZIL: Campos Basin–Holotype–-19.87264444°S -39.99265000°W, 26 m, 17/12/2010, one ind., (MNRJP-003017);–Paratypes–-19.79245278°S -39.72085556°W, 34 m, 14/12/2010, one ind., (MNRJP-003018); -19.95913611°S -39.89185833°W, 43 m, 16/12/2010 one ind., (MNRJP-003019).

Diagnosis. Dorsal tentacles on posterior margin of peristomium. First pair of branchiae on the posterior margin of peristomium, posterior-lateral to tentacles, second pair located in first setiger. Thoracic region slightly expanded with 10–13 setigers and dorsal groove. Abdominal parapodia modified with 6–8 serrated notopodial setae and 8–10 serrated neurosetae capillary setae per segment. Pre-pygidial segments expanded with ventral groove.

**Fig 12:**
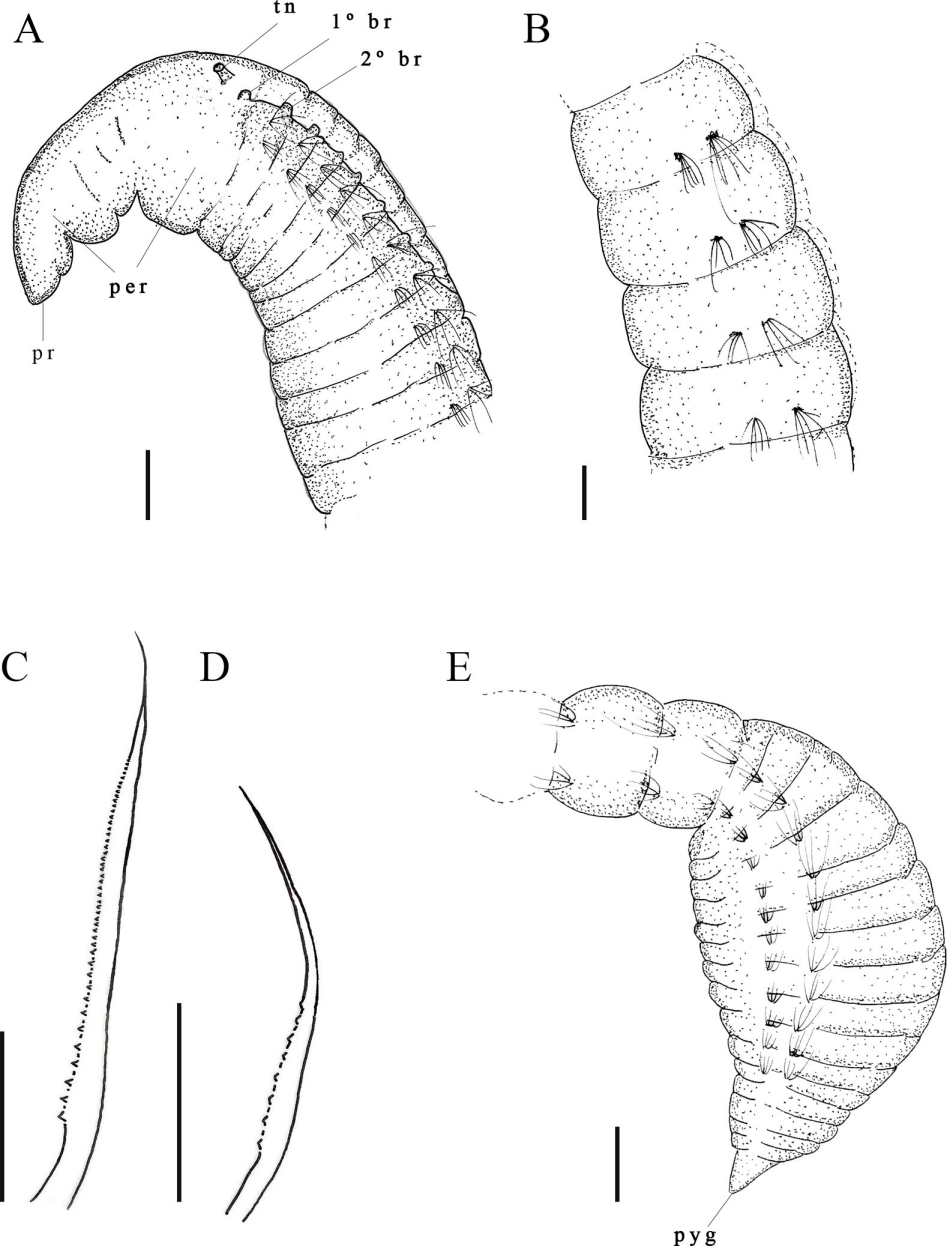
*Kirkegaardia zafirae* sp. nov. (A) anterior end, lateral view; (B) abdominal segments, lateral view; (C) neuropodial setae in posterior region; (D) notopodial setae in posterior region; (E) posterior parapodia, pre-pygidial region. A–E (MNRJP-003017). Scale bars: 100 μm (A-E). Type locality. Atlantic Ocean: Campos Basin (Brazil). Habitat. Fine sediments, sand with biodebris, 26 to 43 m depth. Distribution. Presently known only from Atlantic Ocean: Campos Basin (Brazil). Methyl Green. No pattern, stain not retained.

Description. Holotype with 120 setigers; 3.3 mm long, thoracic region 0.11 mm wide and 0.26 mm high, and abdominal region 0.13 mm wide. Prostomium conical and broad (Fig 12A). Eyes absent. Peristomium elongated, with lateral 2–3 annular grooves, not crossing dorsal surface (Fig 12A). Dorsal tentacles on posterior region of the peristomium. First pair of branchiae in final portion of peristomium, posterior-lateral to tentacles, second pair located in first setiger; branchiae visible up to posterior abdominal region (Fig 12A). Thoracic region slightly expanded with 10–13 setigers and dorsal groove (Fig 12A). Abdominal region with narrow segments, wider than long (Fig 12B). Parapodia reduced, barely visible in optical microscopy. Thoracic parapodia with 3–5 smooth capillaries per fascicle. Abdominal parapodia modified with 6–8 serrated notopodial setae and 8–10 serrated neurosetae capillary setae per segment. Abdominal notosetae slightly longer than abdominal neurosetae. Denticulated neurosetae from setigers 20–35 (Fig 12C). Notosetae and neurosetae modified with well-developed denticles along edge (Fig 12C and 12D). Pre-pygidial segments expanded with ventral groove (Fig 12E). Pygidium with a conical ventral lobe (Fig 12E).

Remarks. *Kirkegaardia zafirae* sp. nov. is similar to *K*. *siblina* (Blake, 1996) [2], in that they both have an expanded pre-pygidial region, but *K*. *siblina* has only neurosetae denticulated while *K*. *zafirae* sp. nov. has noto- and neurosetae denticulated. In addition, *K*. *siblina* has a prominent achaetous segment between the peristomium and the setiger 1, and *K*. *zafirae* sp. nov. has no achaetous segment. *Kirkegaardia zafirae* sp. nov. belongs to the *Kirkegaardia baptisteae*-*tesselata* group due to their similar morphology, species in this group have elevated thoracic parapodia, and a mid-dorsal channel is not produced, although a dorsal crest sometimes develops. In the pre-setigerous area, dorsal ridges and rings are present or absent. From the group *K*. *baptisteae* was the only species that has denticulated notosetae, *K*. *zafirae* sp. nov. differs from *K*. *baptisteae* in that its pre-pygidial region is not flattened ventrally. For all these differences, *K*. *zafirae* sp. nov. was considered as new to science.

Etymology. This species is named in honor of Dr. Zafira da Silva de Almeida for her valuable contribution to marine biodiversity research and the conservation of aquatic resources in Brazil.

Key to species of *Kirkegaardia* found in Brazilian waters.

**1.** Peristomium with up to four annulations…………………………………………………………………**2**

- Peristomium smooth without annulations…………………………………………………………………………………**9**

**2.** Presence of peristomial crest along the peristomium………………………………**3**

- Peristomial crest absent along the peristomium……………………………………..**6**

**3.** Modified setae starting from anterior setigers up to 17 setiger………. . .…………**4**

- Modified setae starting in posterior setigers from 46 setiger………………………..**5**

**4.** Thoracic region with dorsal and slight ventral groove; notopodial modified setae from setigers 10–12; Pre-pygidial region expanded with ventral groove……………………………………….………***Kirkegaardia papaveroi* sp. nov.**

**-** Thoracic dorsal groove present; Notopodial modified setae from setigers 17; Pre-pygidial region expanded, ventral groove absent; Presence of a smaller segment demarcating the beginning of the pre-pygidial region…………………………………………………………………………***Kirkegaardia jongo* sp. nov.**

**5.** Prostomium triangular; Thoracic crest narrow; Neuro and notosetae denticulate present from the setiger 50………………………………………………………………***Kirkegaardia hampsoni***

- Prostomium short and conical; Thoracic crest wide, expanded proximally; Neurosetae denticulated appears from the abdominal setigers 46 to 54……………………………………………………………………………………***Kirkegaardia goytaca* sp. nov.**

**6.** First pair of branchiae postero-lateral to the tentacles in the final portion of the peristomium……………………………………………………………………………………………………**7**

- First pair of branchiae on the first setiger; thoracic groove present; pre-pygidial region slightly expanded with long setae with an inflated tip and setae denticulated……………………………………………………………***Kirkegaardia blakei* sp. nov.**

**7.** Thoracic region groove presence……………………………………………………………………**8**

- Thoracic region groove absence; Thoracic parapodia with 5–7 noto and neurosetae capillary with fibrils per fascicle; Posterior abdominal parapodia with noto- and neurosetae denticulated; Region pre-pygidial extremely expanded…………………………………………………………………………………***Kirkegaardia helenae* sp. nov.**

**8.** Notosetae and neurosetae modified with well-developed denticles along edge; Pre-pygidial segments expanded with ventral groove……………………………………………………………………………***Kirkegaardia zafirae* sp. nov.**

- Notopodial setae capillary, multidentate or with fibrils; Neurosetae modified from setigers 18–32; Posterior abdominal parapodium with 3–5 noto and neurosetae denticulated per fascicle; Pre-pygidial region slightly expanded; Pygidium formed by an enlarged ventral lobe ………………………………………………………………………***Kirkegaardia brisae* sp. nov.**

**9.** Peristomial crest throughout the peristomium, Notopodial setae simple capillary and multidentate only………………………………………..***Kirkegaardia nupem* sp. nov.**

- Peristomial crest absent; notopodial setae with fibrils only……………………………………………………….***Kirkegaardia medusa* sp. nov.**

## Discussion

According to Blake [22], the genus *Kirkegaardia* consists of at least three distinct groups of species and several outlier species for which relationships have yet to be defined. The three main groups are *Kirkegaardia dorsobranchialis*-*heterochaeta* (16 species); *Kirkegaardia baptisteae*-*tesselata* (23 species); and *Kirkegaardia luticastella* group (4 species), in addition to outlier species, with characters of other genera or species not fully characterized (5 species). The species described here belong to the groups of *Kirkegaardia dorsobranchialis*-*heterochaeta* and *Kirkegaardia baptisteae*-*tesselata*.

The species of the group *Kirkegaardia dorsobranchialis*-*heterochaeta* have thoracic parapodia elevated producing a channel between the notopodia, elongate pre-setigerous area that is either entirely smooth or modified with a dorsal ridge and/or rings and denticulated capillaries in both the noto- and neuropodia [22]. This group may be further divided into four subgroups based on peristomial morphology: **(1)** Peristomium smooth, without dorsal ridge; **(2)** Peristomium elongate, smooth with a peristomial ridge limited to the anterior half; **(3)** Peristomium with a dorsal ridge along its entire length; this study adds to this subgroup the species *K*. *papaveroi* sp. nov., *K*. *goytaca* sp. nov. and *K*. *jongo* sp. nov; **(4)** Peristomium unusually long, lacking a dorsal ridge and with 5–6 rings. Although *K*. *blakei* sp. nov. and *K*. *brisae* sp. nov. exhibit 3–4 rings only, we suggest that these species are more comparable to the ones of the latter subgroup 4, but the number of peristomial rings is expanded to 3–6.

The group of *Kirkegaardia baptisteae*-*tesselata* includes species that do not have thoracic parapodia elevated, and a mid-dorsal channel is not produced, although a dorsal ridge is sometimes developed. In the pre-setigerous area, dorsal ridges and rings are present or absent. Most species in this group have denticulations on the neurosetae but not on the notosetae. Those characters are seen in *K*. *nupem* sp. nov., *K*. *helenae* sp. nov., *K*. *medusa* sp. nov., and *K*. *zafirae* sp. nov.. Despite that we categorize the species described herein in these groups, it is important to emphasize the need for in-depth studies on the phylogeny of Cirratulidae and *Kirkegaardia*, so that these groups and their synapomorphies can be well defined.

In this study, the use of the methyl green stain has not shown to be a decisive tool for the identification of morphological characters, as none of the described species showed a well-marked colour pattern among the specimens (S1 Fig). Several studies have pointed to methyl green staining as a very useful tool for taxonomic identification of different families of polychaetes, such as Capitellidae, Cirratulidae, Paraonidae, Sabellidae and Spionidae [39–41]. However, the use of this technique for taxonomy requires standard methodological procedures, for example, the time of exposure to MG, the concentration of the MG solution, and washing times to remove excess MG, so we can be sure that the patterns found in each species or group do not differ due to differences in the methodologies applied for staining.

The present study firstly described ten species of *Kirkegaardia* from Brazil, expanding the number of records for the genus in the coast and deep sea of South America. With the nine new species described in this article, the number of *Kirkegaardia* species has increased from 41 to 50, becoming now one of the most speciose genera of Cirratulidae. In South America, *Kirkegaardia morae* (Elias, Rivero & Orensanz, 2017) [28] was the only registered species before the new species and new records presented here. Additionally, we report a new occurrence for the South Atlantic Ocean (*Kirkegaardia hampsoni*).

## Supporting information

S1 Fig Methyl green staining patterns. (A) *Kirkegaardia blakei* sp. nov.; (B) *Kirkegaardia blakei* sp. nov.; (C) *Kirkegaardia brisae* sp. nov.; (D) *Kirkegaardia brisae* sp. nov., abdominal setigers; (E) *Kirkegaardia helenae* sp. nov.; (F) *Kirkegaardia helenae* sp. nov.; (G) *Kirkegaardia nupem* sp. nov.; (H) *Kirkegaardia nupem* sp. nov.; (I) *Kirkegaardia goytaca* sp. nov., thoracic region; (J) *Kirkegaardia medusa* sp. nov.; (K) *Kirkegaardia jongo* sp. nov.
(TIF)
