# Supplementary figures and images for: Kirkegaardia Blake, 2016 (Annelida: Cirratulidae) from Southeastern Brazil with description of nine new species
Source: PLoS One. 2022 May 10;17(5):e0265336. doi: 10.1371/journal.pone.0265336 (PMC9090474; doi:10.1371/journal.pone.0265336)

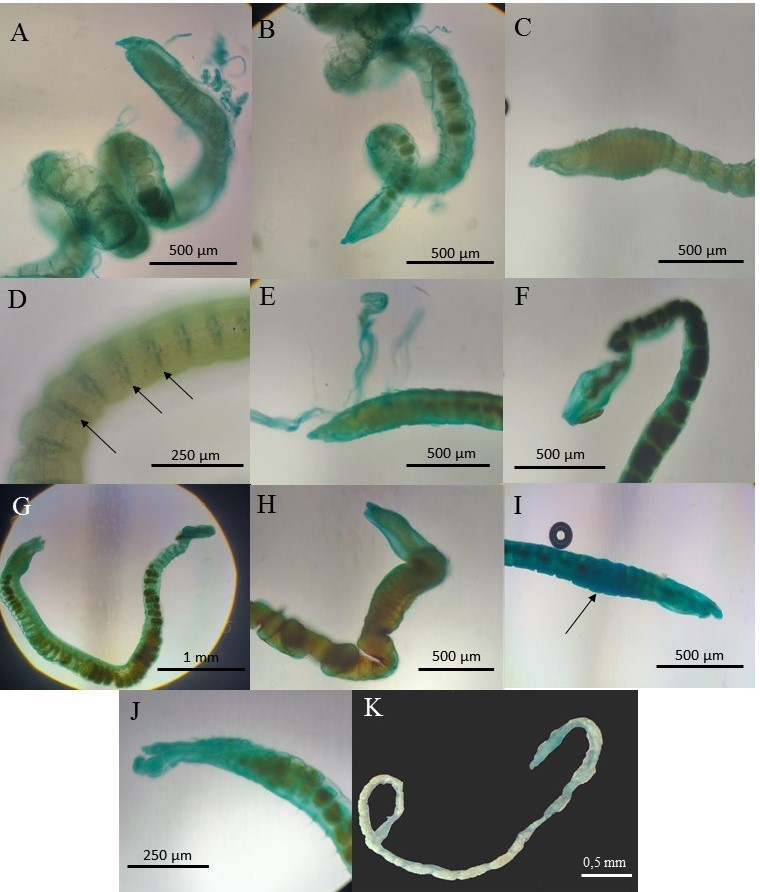

Supplement: S1 Fig — (A) Kirkegaardia blakei sp. nov.; (B) Kirkegaardia blakei sp. nov.; (C) Kirkegaardia brisae sp. nov.; (D) Kirkegaardia brisae sp. nov., abdominal setigers; (E) Kirkegaardia helenae sp. nov.; (F) Kirkegaardia helenae sp. nov.; (G) Kirkegaardia nupem sp. nov.; (H) Kirkegaardia nupem sp. nov.; (I) Kirkegaardia goytaca sp. nov., thoracic region; (J) Kirkegaardia medusa sp. nov.; (K) Kirkegaardia jongo sp. nov. (TIF) [file pone.0265336.s001.tif]
